# Multimodal deep learning improving the accuracy of pathological diagnoses for membranous nephropathy

**DOI:** 10.1080/0886022X.2025.2528106

**Published:** 2025-07-14

**Authors:** Xiuxiu Hu, Jinyue Yang, Yiping Li, Yuxiang Gong, Haifeng Ni, Qing Wei, Minyu Yang, Yu Zhang, Jing Huang, Cao Ma, Bizhen Wei, Kaijie Yu, Jiayun Xu, Siyu Xia, Taotao Tang, Pingsheng Chen

**Affiliations:** a Department of Pathology, School of Medicine, Southeast University, Nanjing, China; b School of Automation, Southeast University, Nanjing, China; c Department of Nephrology, Zhongda Hospital, Southeast University, Nanjing, China; d Department of Respiratory and Critical Care Medicine, Zhongda Hospital, Southeast University, Nanjing, China; e Department of Nephrology, The First Affiliated Hospital of Henan University of Science and Technology, Luoyang, China

**Keywords:** Membranous nephropathy, renal biopsy, multimodal pathological diagnosis, artificial intelligence, deep learning

## Abstract

**Objectives:**

Renal biopsy is the gold standard for the diagnosis of glomerular diseases including membranous nephropathy (MN), however, it faces challenges in accuracy, objectivity, and reproducibility of tissue evaluation. This study aims to develop a multimodal pathological diagnosis system to assist pathologists in diagnosing MN in morphology.

**Methods:**

Using PASM-stained, immunofluorescence, and electron microscopy images from MN patients, we built three deep-learning models to detect lesions. The outputs of these models were combined to provide a comprehensive pathological diagnosis. Our system was compared with pathologists, validated on external test sets, and detected in 138 patients with various kidney diseases.

**Results:**

Considering PASM-stained images, our model had a classification accuracy of 91.74%, a recall of 81.97%, and an F1 score of 86.58% for spike identification. For immunofluorescence images, our model had an accuracy rate of 98.97%, a recall rate of 99.65%, and an F1 score of 99.31% for MN classification. Regarding the segmentation of electron-dense deposits, the segmentation model had a Dice coefficient of 85.66% and an IoU of 75.93%. Our model presented superior performance to that of pathologists in fluorescence image classification and segmentation of deposits, achieved high accuracy in spike identification and fluorescence image classification in external test sets, and could be targeted to diagnose MN in a wide range of glomerular diseases.

**Conclusions:**

This multimodal pathological diagnosis system can not only assist pathologists in diagnosing MN rapidly and accurately but also lays the foundation to develop diagnostic models for other glomerular diseases.

## Introduction

Membranous nephropathy (MN) is a common pathological type of primary glomerular disease that can lead to adult nephrotic syndrome [1–3]. Approximately 30% of patients progress to chronic kidney disease and end-stage renal disease, so an accurate diagnosis of MN is critical to optimize medical management and improve prognosis [4,5]. Although nephrotic syndrome can be initially diagnosed by clinical manifestations and serum and urine indices, the definitive diagnosis of MN must rely on renal biopsy [6,7]. However, renal biopsy is both expensive and time-consuming, and a series of problems such as the severe shortage of renal pathologists and poor diagnostic consistency among pathologists have brought great challenges to the diagnosis of renal diseases [8,9]. Therefore, it is clinically important to establish a more objective and accurate method for diagnosing and evaluating MN from a pathological perspective.

Significant progress and breakthroughs have been made in deep learning (DL) medical images recently, and driven by advances in computer technology [10,11]. The convolutional neural network (CNN) is an important branch of DL that has been applied to a variety of computer vision tasks, such as image classification, target detection, and image segmentation in the diagnosis and treatment of many kinds of diseases [12–14]. Recently, the development of multimodal and large models in digital pathology has been driven by the construction of extensive datasets and advancements in modeling architectures. Firstly, large-scale pre-training combined with contextual modeling has significantly enhanced the accuracy of pathological diagnosis and improved model generalization, laying a critical foundation for precision medicine [15]. Secondly, the integration of advanced pre-training strategies has substantially increased the efficiency of feature extraction from pathological images, leading to notable success in cancer detection and prognosis prediction, particularly in pan-cancer screening and the diagnosis of rare cancers [16,17]. DL is advancing rapidly across medical fields such as oncology, retinal diseases, cardiovascular disorders, neurodegenerative conditions, and diabetes, propelled by significant progress in multimodal and large-scale modeling approaches [18–22]. These advancements not only validate the profound potential of DL in the realm of digital pathology but also establish a solid foundation for the development of efficient and accurate diagnostic tools. However, the application of DL in kidney disease remains relatively underdeveloped. While pathologists have utilized DL models to evaluate kidney tissue lesions, identify glomeruli, and classify kidney diseases [23–25], the inherent diversity and complexity of kidney pathologies continue to pose significant challenges. The application of multimodal and large-scale modeling technologies in kidney disease research holds the potential to overcome existing bottlenecks, enabling more accurate diagnoses, personalized treatment strategies, and improved disease prognostication.

Therefore, this study aims to build an efficient and accurate pathological diagnosis system for MN. The system is based on three types of renal biopsy pathology images and employs multimodal DL models to achieve pathological diagnoses of MN. The diagnostic system comprises three DL models: (1) a spike classification model for glomeruli based on periodic acid-silver methenamine (PASM)-stained images, (2) a fluorescence classification model for renal diseases based on immunofluorescence (IF) images, and (3) a deposits segmentation model for electron-dense deposits based on electron microscopy images. This approach will assist nephrologists to more accurately diagnose MN from a pathological perspective while laying the groundwork to develop diagnostic models for other types of glomerular diseases.

## Methods

### Dataset collection

Renal biopsy samples were collected from patients diagnosed with idiopathic MN between March 2018 and December 2020 at the Institute of Nephrology, Southeast University. Inclusion criteria: (1) age at least 18 years, (2) glomerular filtration rate (GFR) ≥30 mL/min/1.73 m^2^, (3) 24-h urine protein excretion ≥3.5 g, and (4) primary MN confirmed by renal biopsy. Exclusion criteria: (1) secondary MN caused by systemic diseases (e.g., systemic lupus erythematosus), infections (e.g., hepatitis B, hepatitis C), drugs (e.g., gold agents, penicillamine) or tumors, (2) Comorbid with other types of renal diseases (e.g., glomerulopathies, chronic kidney diseases), and (3) severe renal insufficiency: GFR < 30 mL/min/1.73 m^2^ or requiring dialysis treatment, (4) Comorbid with other serious diseases (e.g., severe cardiovascular, hepatic, pulmonary, or other systemic diseases), (5) drug user: recent use of medications related to weakened renal function and proteinuria, such as immunosuppressants (used within three months before study entry), (6) pregnant or lactating women.

MN is characterized by glomerular subepithelial immune complex deposition, which can be visualized by PASM-stained, Immunoglobulin G (IgG) IF staining (1:80, ZSGB-BIO), and immunoelectron microscopy images. Therefore, the above three types of images were used to establish DL models. In addition, we collected immunoglobulin A -stained (1:80, ZSGB-BIO) images of patients with IgA nephropathy (IgAN) and IgG-stained (1:80, ZSGB-BIO) IF images of patients with diabetic nephropathy (DN) to highlight the differences in IF features between MN and the two kinds of renal diseases, mainly in location and expression pattern of positive expression regions of fluorescent staining. This study primarily focused on the pattern and location of immune complex deposition, emphasizing qualitative diagnosis rather than quantitative analysis. Therefore, fluorescence intensity was not included in the study. The reference standards for immunofluorescence images were based on the immune deposition patterns described in the KDIGO 2021 clinical practice guidelines for glomerular diseases, as well as the immune complex classification criteria outlined in the ISN/RPS 2018 guidelines for lupus nephritis [26,27].

In this study, both PASM-stained images and IF images were taken with an Olympus BX41 light microscope, and the magnification factor is 400× and 200×, respectively. Electron microscope images were obtained *via* an HT7700 transmission electron microscope at magnifications ranging from 3,000× to 5,000×. The external test set 1 included the images of MN from the Institute of Nephrology, Southeast University 2024. The external test set 2 comprised the figures from the Department of Nephrology, the First Affiliated Hospital of Henan University of Science and Technology. Meanwhile, to differentiate MN from other glomerular diseases, we collected the same types of images from the patients with MN, IgAN, DN, lupus nephritis (LN), and minimal change disease (MCD) in our hospital.

### Image preprocessing

**Figure 1.**
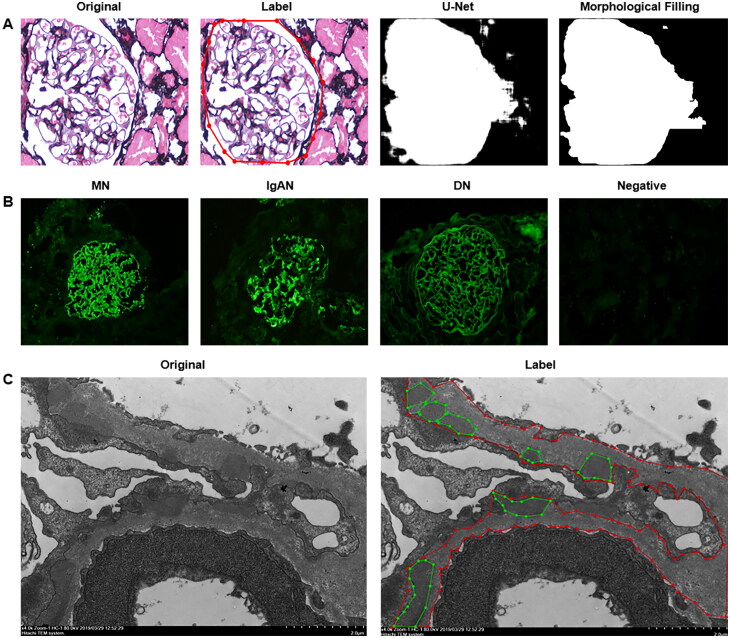
Pretreatment method of image classification and segmentation. (A) Segmentation of glomeruli. The pathologists labeled the glomerulus (red closed ring), U-net segmented the glomerulus (white for the glomerular outline, black for the background) and subsequent morphological filling made the glomerular outline clearer. The magnification was 400×. (B) Classification of fluorescent images. Pathologists classified the fluorescence images according to the characteristics of the lesions. Fluorescence in MN, IgAN, and DN were immunoglobulin G (IgG) granular deposition along glomerular capillary loops, immunoglobulin A (IgA) deposition in the mesangial region, and IgG linear deposition along the glomerular capillary loops, respectively. The magnification is 200×. (C) Labeling of electron-dense deposit. The pathologists marked the outlines of the basement membrane and the deposits. The red areas indicate glomerular basement membranes and the green areas for the electron-dense deposits. MN: membranous nephropathy; IgAN: IgA nephropathy; DN: diabetic nephropathy.

PASM-stained images were initially classified into two groups: lesion group and no-lesion group based on lesion characteristics. Subsequently, color normalization was performed, and the images were segmented into glomeruli using U-Net and morphological fill. The segmented glomerular images were divided into thousands of patches, which were used for final classification. Fluorescence image classification was performed by pathologists and used directly to train the model without preprocessing. The preprocessing to segment electron-dense deposits was to label the glomerular basement membrane and electron-dense deposits using labeling software by pathologists. The detailed preprocessing method was shown in Figure 1. All three pathological images were reviewed back-to-back by three pathologists, and images with consistent reviews were selected for inclusion in the dataset for study. Additionally, to enrich the experimental data and enhance the generalization ability of the model, the study also applied mirror flipping, random rotation, and random cropping data enhancement algorithms.

### Establishment of the pathological diagnosis system

**Figure 2.**
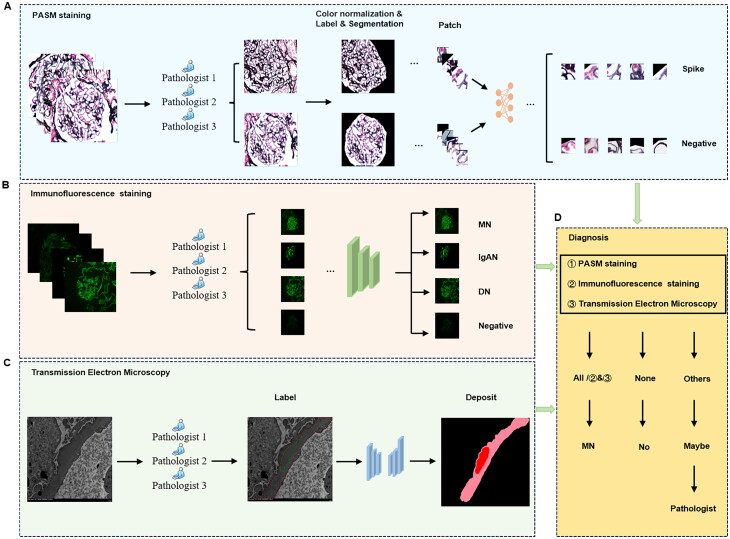
The overall workflow for the diagnostic of MN. (A) Classification of spikes. Pathologists classify PASM images with and without spikes based on lesion characteristics, then color normalizes the images, and manually label and segment glomeruli using U-Net. Finally, the glomeruli are sliced into thousands of patches and the model detects spikes on the patches. (B) Pathologists group images based on the fluorescence features of different diseases and build deep learning fluorescence classification models to identify and classify the fluorescence features. (C) Pathologists label basement membranes and electron-dense deposits to establish a segmentation model, after which electron microscopic images of MN could be input into the model to segment the deposits. (D) Combining the results of the three models to diagnose MN. If all three conditions are met, MN can be diagnosed; if none of them are met, MN is excluded; if they are partially met, MN is suspected, and the diagnosis can be done under the supervision of pathologists. MN: membranous nephropathy; IgAN: IgA nephropathy; DN: diabetic nephropathy; PASM: periodic acid-silver methenamine.

The pathological diagnosis system for MN consists of four parts: (A) constructing a CNN using PASM-stained images of MN to identify glomerular spikes; (B) constructing a CNN using IF images of four kidney diseases to classify fluorescence images of kidney diseases; (C) creating a CNN using electron microscope images to segment the subepithelial electron-dense deposits of the glomerular basement membranes and (D) combining the results of the above three parts to make a diagnosis. The overall flow chart is shown in Figure 2.

### Classification model for glomerular spikes

We proposed a CNN to classify the images stained with PASM from MN patients based on the lesion characteristics of diffuse spikes in the basement membrane of glomerular capillaries loops. The residual network ResNet was used as a framework backbone network to which the contextual self-attention module and cross-convolution were added [28], the network structure is shown in Supplementary Figure 1.

### Classification model for fluorescence images

IF images of four kinds of renal diseases were gathered and classified according to fluorescent lesion features. A CNN with dense connections based on ResNeXt was designed to improve the classification of different fluorescence images [29]. The overall network structure is shown in Supplementary Table 1. Every IF pathological image involved in the study included at least one glomerulus, therefore, we distinguished different types of fluorescence images by the shape and location of immunocomplex.

### Segmentation model for electron-dense deposits

MN is characterized by the deposition of electron-dense deposits under the glomerular epithelium in electron microscopic lesions. On this basis, we proposed a network framework based on a ‘U’-shaped encoder and decoder, to which we added a multiscale connectivity module and a channel attention that further highlights the important features while extracting local details and global semantic information at all scales. As shown in Supplementary Figure 2, the network employed electron microscopy images of the glomerular basement membrane as input signals. Initially, the encoder was responsible for extracting the image features, and the decoding path was symmetric with the encoding path. Subsequently, the decoder recovered the size of the electron microscopy feature maps and performed pixel classification of electron-dense deposits in the basement membrane. Ultimately, the pathologist diagnosed MN based on the final segmented images of the deposits.

### Pathologist comparison, external validation and comprehensive diagnosis

To demonstrate the accuracy of our model, three pathologists were asked to evaluate the test set images and compare them with the corresponding real labels, and assess the diagnostic consistency of the model-pathologists and inter-pathologists. Furthermore, we investigated the relationship between the segmentation model’s performance and the consistency of the pathologists’ evaluations. In addition, we collected three types of renal biopsy images from two external test sets to demonstrate the generalization performance of the model. The images of the two external sets were from different datasets and prepared inconsistently in terms of the staining reagents, sample preparation technicians, and pathologists, which could better detect the generalization capability of our DL models. In the meantime, three types of biopsy images from 43 patients with MN, 40 with IgAN, 18 with DN, 20 with LN, and 17 with MCD were collected and entered into the system for recognition to evaluate the accuracy of the diagnostic system.

### Implementation details

We have developed three independent DL models for PASM-stained images, IF images, and electron microscope images, respectively, to accommodate differences in image resolution, structural features, and diagnostic tasks. Each model adopted a modality-specific network architecture. The key training configurations are summarized in Supplementary Table 2, and network structures are illustrated in Supplementary Figures 1, Supplementary Table 1, Supplementary Figures 2. All models were implemented in PyTorch 1.2.0 and trained on an NVIDIA GTX 2080Ti GPU using Python 3.6 and CUDA 10.0.

### Evaluation of model performance

First, we constructed a confusion matrix based on various matches between prediction and ground truth as shown in Supplementary Table 3. Three indicators, precision, recall, and F1 were calculated to evaluate the performance of the glomerular spike and fluorescence image classification models.

The formula for each evaluation indicator is as follows.

(1) Precision
  (1) $$\text{Precision}=\frac{\text{TP}}{\text{TP}+\text{FP}}$$
(2) Recall
  (2) $$\text{Recall}=\frac{\text{TP}}{\text{TP}+\text{FN}}$$
(3) F1 score
  (3) $$F1=\frac{2\times P\times R}{P+R}$$

For fluorescence image classification, the evaluation metrics include the macro-precision rate (macro-P), the macro-recall rate (macro-R) and macro-F1 (macro-F1).

(4) Macro-P
  (4) $$\text{macro}-P=\frac{1}{n}\sum_{1}^{n}p_{i}$$

In the above formula, n denotes the total number of fluorescence image categories and p*_i_* denotes the precision rate of samples in each category. p*_i_* is calculated according to the following formula.

(5) $$p_{i}=\frac{{\text{TP}}_{i}}{{\text{TP}}_{i}+{\text{FP}}_{i}}$$

(5) Macro-R
  (6) $$\text{macro}-R=\frac{1}{n}\sum_{1}^{n}r_{i}$$

In the above formula, n denotes the total number of fluorescence image categories and r*_i_* denotes the recall of samples in each category. r*_i_* is calculated as follows.

(7) $$r_{i}=\frac{{\text{TP}}_{i}}{{\text{TP}}_{i}+{\text{FN}}_{i}}$$

(6) Macro-F1
  (8) $$\text{macro}-F1=\frac{2\times \text{macro}-P\times \text{macro}-R}{\text{macro}-P+\text{macro}-R}$$

For electron microscope images, the evaluation metrics include Dice similarity coefficients, Intersection over Union (IoU) intersections.

(7) Dice

The Dice similarity coefficient is a set similarity measurement index used to calculate the degree of similarity between two samples. The larger the value, the higher the similarity. The calculation formula is:

(9) $$\text{Dice}=\frac{2\text{TP}}{\text{FP}+2\text{TP}+\text{FN}}$$

(8) IoU

IoU refers to the degree of overlap between the predicted area of the model and the truly labeled area. The calculation formula is:

(10) $$\text{IOU}=\frac{\text{TP}}{\text{TP}+\text{FP}+\text{FN}}$$

### Statistical analysis

Cohen’s kappa coefficient was used to evaluate the agreement between the model and individual pathologists, as well as among the pathologists, in spike identification and IF classification. For the segmentation of electron-dense deposits, the intraclass correlation coefficient (ICC) was applied to assess the consistency of annotations. To explore the potential impact of inter-pathologist annotation variability on model performance, the standard deviations of Dice and IoU scores among the three pathologists were calculated for each image using Pearson correlation analysis. All statistical analyses were conducted using R software (version 4.5.0).

## Results

### Dataset distribution

We collected PASM-stained images, IF images, and electron microscopy images for the diagnosis of MN, as well as IF images for IgAN and DN, and fluorescence-negative images. The distribution of all the images included in the study is shown in Table 1.

**Table 1.** Distribution of datasets for three renal biopsy images.

Picture type	Train & validation	Test	External 1	External 2
PASM				
Spikes	510	365	88	98
Negative	1,275		91	108
IF				
MN	578	289	230	203
IgAN	489	164	136	106
DN	322	81	92	153
Negative	384	129	175	128
TEM	999	144	199	133

*Note:* PASM: periodic acid-silver methenamine; IF: immunofluorescence; TEM: transmission electron microscopy; MN: membranous nephropathy; IgAN: IgA nephropathy; DN: diabetic nephropathy.

### Classification of spikes

In MN, PASM-stained images characteristically show diffuse spikes in the glomerular capillary basement membrane. Based on the properties of spikes, the classification network provided a precision rate of 91.74%, a recall rate of 81.97%, and an F1 of 86.58%. Meanwhile, the proposed model was compared with the classification methods used in other related research, and the results of each algorithm are shown in Table 2. Our image classification network model achieved optimal results in the spike classification, with the maximum values of all three metrics. In comparison to the backbone network, our model demonstrated an improvement of 1.84% in precision and 9.02% in recall. To further prove the effectiveness of the algorithm, we conducted ablation experiments for the contextual self-attention module and cross-convolution. As shown in Supplementary Table 4, the contextual self-attention module and cross-convolution could help to correctly categorize spikes.

**Table 2.** Comparison results of our model with other classification methods related to spike identification.

Method	Precision (%)	Recall (%)	F1(%)
InceptionV3	86.30	51.64	64.62
GoogLeNet	82.09	45.08	58.20
VGG	74.19	37.7	49.99
ResNet18	77.19	36.07	49.17
ResNet34	85.00	55.74	67.33
ResNet50	88.10	30.33	45.12
ResNet101	89.90	72.95	80.54
**Cross-CoTNet (Ours)**	**91.74**	**81.97**	**86.58**

*Note:* ResNet101: backbone network.

The performance comparison between our proposed model and other models across different tasks. The bold values indicate the best results achieved under each evaluation metric.

**Figure 3.**
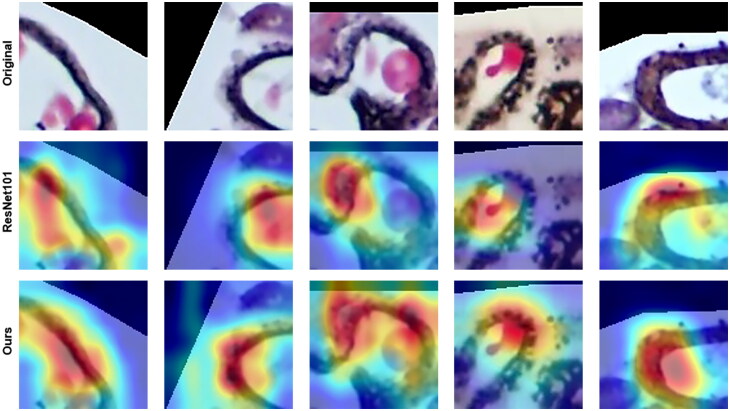
Visualization of spikes in the glomerulus. These patch images with 400× magnification are PASM-stained renal tissue after network cutting. The original patches show the distribution of spikes along glomerular capillary loops, the ResNet101 algorithm only focuses on part of the diseased area, and our algorithm accurately predicts the location of spikes. (ResNet101 is one of the ResNet series of residual networks, which is the backbone network of this study. Colors represent areas of network interest, decreasing in importance from red to blue).

Further validation of our model was performed by generating a visual interpretation of the classification results. As shown in Figure 3, the ResNet101 algorithm only focused on part of the lesion area and did not strictly detect the area along the glomerular capillary loops. However, our algorithm enhanced the extraction and utilization of texture information, and the detected spikes more closely match the lesion range, reducing the probability of missed detection. Furthermore, the network strengthened the learning of neighboring features and was available to expand basically along the grain of the capillary wall based on the lesion area, which also verified the validity of cross-convolution.

### Fluorescence image classification

The fluorescence images of different renal diseases exhibit distinct distribution characteristics, as shown in Figure 1(B). Regarding the most dominant expression mode in the glomerular capillary loops, MN displays granular deposition of IgG, IgAN presents granular or clumped deposition of IgA, and DN shows linear deposition of IgG. Compared with the currently used algorithm models to classify renal pathological fluorescence images, the model constructed in this study had better performance. As shown in Table 3, our model achieved a high degree of accuracy in the classification task for four types of fluorescent images, with a macro precision of 96.86%, a macro recall of 97.4%, and a macro F1 score of 97.13%. When compared to the GoogLeNet model, which similarly employed a multi-branch convolutional architecture, our model demonstrated a remarkable improvement of over 10% across three pivotal metrics: macroscopic precision, macroscopic recall, and macroscopic F1 score. Furthermore, the present model exhibited enhanced classification efficacy compared to the DenseNet model with dense connectivity characteristics. These findings substantiated the efficacy of the strategy of combining multi-branch convolution with dense connectivity. Meanwhile, the proposed algorithm achieved a precision of 98.97%, a recall of 99.65%, and an F1 score of 99.31% for the representative images of MN.

**Table 3.** Comparison results of fluorescence classification models.

Method	Macro-P (%)	Macro-R (%)	Macro-F1 (%)	Precision (%)		Recall (%)		F1(%)	
				MN	Negative	MN	Negative	MN	Negative
ResNet50	73.76	62.33	67.57	68.78	78.85	97.58	63.57	80.69	70.39
ResNet101	92.94	93.52	93.23	97.90	88.32	96.89	93.80	97.39	90.98
GoogLeNet	82.85	83.43	83.14	94.96	77.78	91.35	92.25	93.12	84.40
Densenet121	95.40	95.65	95.52	98.62	93.70	98.96	92.25	98.79	92.97
Densenet169	94.43	94.17	94.30	97.04	89.63	97.04	93.80	97.04	91.67
ResNeXt101_32 × 8d	93.77	94.02	93.89	97.90	89.05	96.89	94.57	97.39	91.73
ResNeXt101_32 × 16d	94.91	95.36	95.13	97.92	92.42	97.92	94.57	97.92	93.48
ResNeXt101_32 × 32d	94.93	95.46	95.19	98.29	95.87	99.31	89.92	98.80	92.80
**D-ResNeXt101_32 × 32d (Ours)**	**96.86**	**97.40**	**97.13**	**98.97**	**97.56**	**99.65**	**93.02**	**99.31**	**95.24**

*Notes:* Negative, fluorescence-negative images in cases of non-MN, IgAN and DN. MN: membranous nephropathy.

The performance comparison between our proposed model and other models across different tasks. The bold values indicate the best results achieved under each evaluation metric.

### Segmentation of electron-dense deposits

Electron microscopic lesions in MN show subepithelial electron-dense deposits on the outer glomerular basement membrane. To construct the segmentation model, pathologists manually labeled the outlines of the basement membrane and electron-dense deposits (Figure 1(C)).

**Figure 4.**
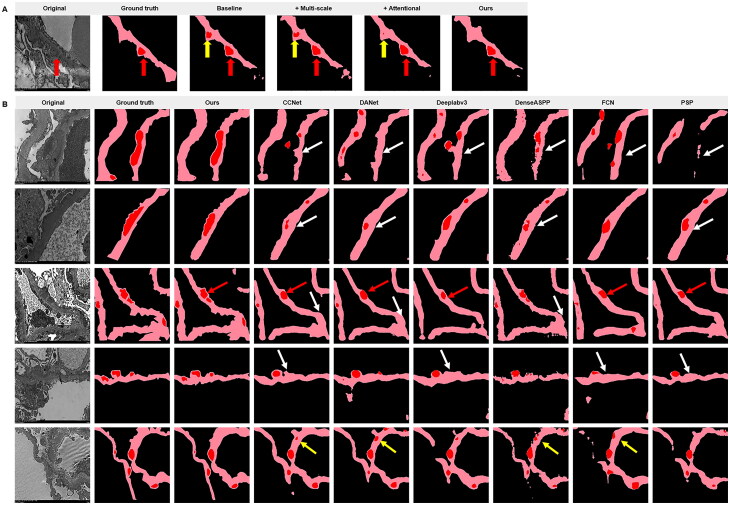
Construction and comparison of segmentation model. (A) Comparison of segmentation results of ablation experiments. Our algorithm with both multiscale connectivity and channel attention produces more accurate segmentation than the models with only multiscale connectivity or channel attention modules (red arrows indicate correctly segmented electron-dense deposits and yellow arrows point to missegmented electron-dense deposits). (B) Segmentation of deposits with different methods. Five cases were illustrated in the segmentation diagram, the black area was the background, and the light pink and red area standed for glomerular basement membrane and deposits, respectively. The ground truth was based on the labeling of pathologists. White arrows and yellow arrows point to miss-segmented and mis-segmented deposits, respectively. FCN: fully convolutional network; PSP: pyramid scene parsing network.

We evaluated the performance of the constructed models as well as several DL models that are currently representative and widely used in medical image segmentation tasks. The results (Table 4) showed that our model could accurately segment electron-dense deposits with a higher Dice coefficient (85.66%) and IoU (75.93%). It suggested that the proposed segmentation model made better performance than those of the other models. Similarly, we also performed ablation experiments to evaluate the effectiveness of multiscale connectivity and channel attention (Figure 4(A) and Supplementary Table 5). While the multiscale connectivity and channel attention each improved the performance of the segmentation network model, the improvement in segmentation model performance was particularly significant when they were used in combination, and the resulting segmentation maps were more accurate than when the two modules were applied individually.

**Table 4.** Comparison results of electron-dense deposit segmentation.

Method	IoU (%)	Dice (%)
CCNet	44.23	57.33
DANet	44.15	57.82
Deeplabv3	39.78	52.97
DenseASPP	44.75	58.93
FCN	34.88	47.80
PSP	40.41	53.03
**AGMS-UNet (Ours)**	**75.93**	**85.66**

*Notes:* FCN: fully convolutional network; PSP: pyramid scene parsing network.

The performance comparison between our proposed model and other models across different tasks. The bold values indicate the best results achieved under each evaluation metric.

Among all the appraisal methods (Figure 4(B)), our model was the closest to the pathologic labeled results in segmenting the glomerular electron-dense deposits of MN. In addition, as shown in Figure 4, our model segmented the deposits even when they were close to each other (red arrows), and was less likely to produce some mis-segmentation (yellow arrows) and miss-segmentation (white arrows) compared to other segmentation models. Therefore, our model could segment glomerular electron-dense deposits of MN more accurately.

### Pathologist comparison and external validation

**Figure 5.**
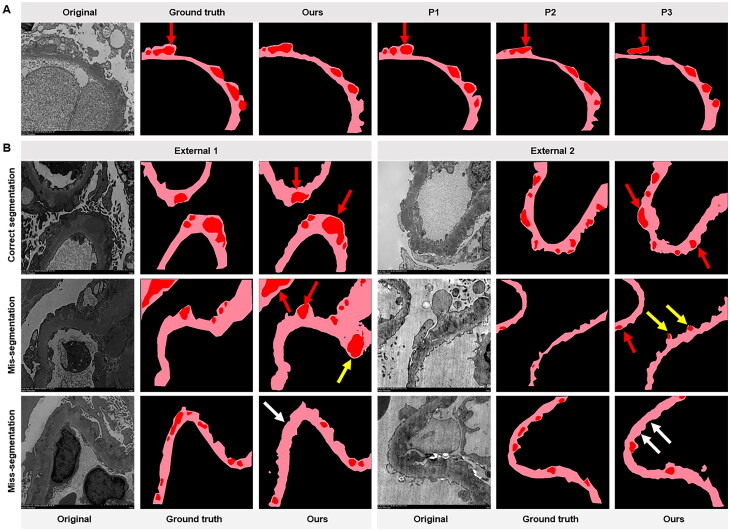
The segmentation of electron-dense deposits in our construction model contrasted three pathologists and different data sets. (A) Deposits and basement membranes are labeled by three pathologists. The red arrows indicate the labeling of deposits. (B) The model can accurately segment the deposits in both external test sets. Red arrows indicate accurately segmentation deposits. Mis-segmentation (false positive) refers to the model missegment other tissues as deposits (indicated by yellow arrows); miss-segmentation (false negative) refers to the model failing to segment deposits (indicated by white arrows); both of these occur when adjacent to the mesangial region, when tissues are deeply stained, and when staining interferes. Ours: our algorithm model; P1: P2, P3: pathologists; E1: external test set 1; E2: external test set 2.

To verify the accuracy and generalization performance of the model, we compared the evaluation results of the model with three pathologists and validated them in two external test sets. As presented in Table 5, the F1 scores for this model were higher than those of pathologist 3 when using the same PASM-stained images to classify spikes. In IF image classification, the model exhibited higher accuracy than the pathologists. The exciting finding was that the model significantly outperformed the pathologists in segmenting the electron microscopy images. Figure 5(A) presented the images labeled by different pathologists, highlighting variations in labeling techniques that led to inconsistent results in deposit segmentation, resulting in poorer performance. In the two external test sets, we observed similar results. It demonstrated that the model performed well in fluorescent image classification, achieving high accuracy, but relatively poorly on the deposit segmentation task. Additionally, in the external test set 2, the model had high accuracy in recognizing spikes. As illustrated in Figure 5(B), mis-segmentation and miss-segmentation tended to occur near the mesangial region and in the more deeply stained regions.

**Table 5.** The performance in our model compared with pathologists and external test sets.

	PASM			IF									TEM	
	Precision (%)	Recall (%)	F1 (%)	Macro-P (%)	Macro-R (%)	Macro-F1 (%)	Precision (%)		Recall (%)		F1(%)		Dice (%)	IoU (%)
							MN	Negative	MN	Negative	MN	Negative		
P1	96.66	100	98.31	93.36	90.62	91.86	92.26	97.41	98.96	87.60	95.49	92.24	63.41	48.26
P2	100	83.90	91.25	92.12	91.97	91.60	93.43	81.01	95.74	99.22	94.57	89.20	41.39	55.67
P3	100	71.26	83.22	95.65	95.30	95.46	96.61	94.66	98.61	96.12	97.60	95.38	51.03	64.95
**Ours**	**91.74**	**81.97**	**86.58**	**96.86**	**97.40**	**97.13**	**98.97**	**97.56**	**99.65**	**93.02**	**99.31**	**95.24**	**85.66**	**75.93**
E1	83.13	78.41	80.70	96.76	97.08	96.92	99.55	98.85	96.96	98.29	97.59	98.57	53.97	69.13
E2	91.09	92.92	92.00	96.96	95.52	96.24	94.42	99.17	100	93.75	97.13	96.39	45.85	32.93

*Note:* PASM: periodic acid-silver methenamine; IF: immunofluorescence; TEM: transmission electron microscopy; Ours: our algorithm model; P1, P2, P3: pathologists; E1: external test set 1; E2: external test set 2.

Compares the diagnostic performance of our model with that of the pathologists, including results on the external test set.

**Figure 6.**
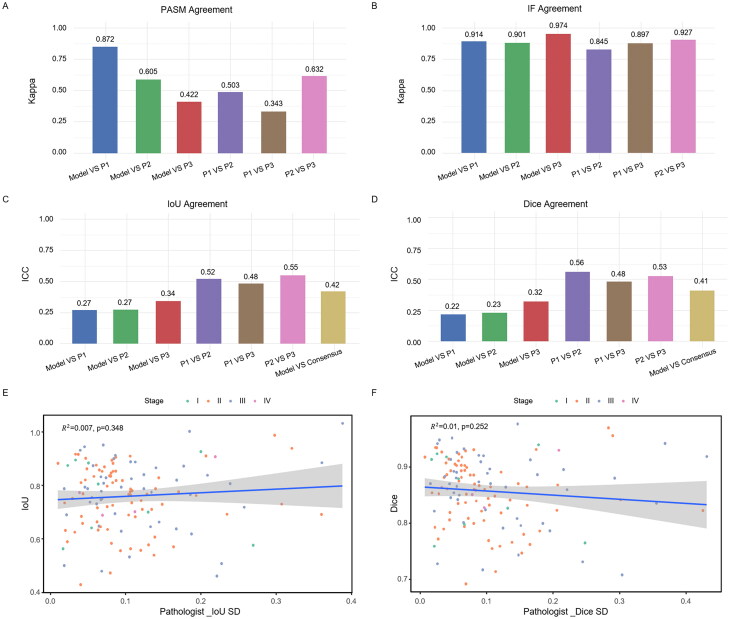
Agreement between model and pathologists and annotation variability in deposit segmentation. (A) Spike identification: the column diagram showing Cohen’s kappa coefficients for spike identification between the model and each pathologist. The high kappa value (κ = 0.872) indicates strong agreement in spike detection. (B) IF classification: the histogram presenting Cohen’s kappa coefficients for agreement between the model and pathologists across major IF staining patterns recognition. The very high kappa value (κ = 0.974) suggests excellent  consistency in IF pattern recognition. (C) Deposit segmentation: IoU-based ICC. ICC analysis based on IoU scores showing comparable agreement between the model and pathologists consensus (ICC = 0.42), demonstrating moderate agreement. (D) Deposit segmentation: dice-based ICC. ICC analysis of Dice scores between the model and pathologists consensus (ICC = 0.41), exhibiting moderate agreement. ICC analysis based on IoU scores showed comparable agreement between the model and pathologists consensus (ICC = 0.42), indicating moderate agreement. (E) Correlation between annotation variability and model IoU scores: the scatter plot illustrating the relationship of IoU performances between the standard deviation of pathologists and the model (R^2^ = 0.007, *p* = .348), indicating minimal influence of annotation inconsistency. (F) Correlation between annotation variability and model Dice scores: the scatter plot showing the relationship of Dice scores between the standard deviation of pathologists and the model (R^2^ = 0.01, *p* = .252), implying limited impact of annotation variability on segmentation accuracy. PASM: periodic acid-silver methenamine; IF: immunofluorescence; IoU: Intersection over Union.

Agreement analyses between the model and the pathologists, as well as among the three pathologists, demonstrated that the model achieved a high level of concordance with pathologists in spike detection, with a Cohen’s kappa value of 0.872 (Figure 6(A)). Even greater agreement was observed in IF classification, with a kappa value of 0.974, and all kappa metrics exceeded 0.84, indicating excellent consistency (Figure 6(B)). For the segmentation of electron-dense deposits, the ICC between the model and pathologist consensus was 0.42 and 0.41 for IoU and Dice, respectively (Figure 6(C,D)). These results illustrated the model’s robustness in tackling complex segmentation tasks and the inherent subjectivity in manual annotations. To assess the potential impact of inter-observer variability on model performance, the correlation between the standard deviation of pathologists’ Dice and IoU scores and the model’s segmentation performance against the consensus was examined. The data demonstrated that the relationship was weak, with an R^2^ of 0.007 (*p* = 0.348) for IoU and R^2^ of 0.01 (*p* = .252) for Dice (Figure 6(E,F)), which suggested that annotation variability among pathologists had minimal influence on the model’s segmentation accuracy, underscoring the model’s stability and reliability in the face of subjective labeling differences.

### Comprehensive diagnoses of MN

**Figure 7.**
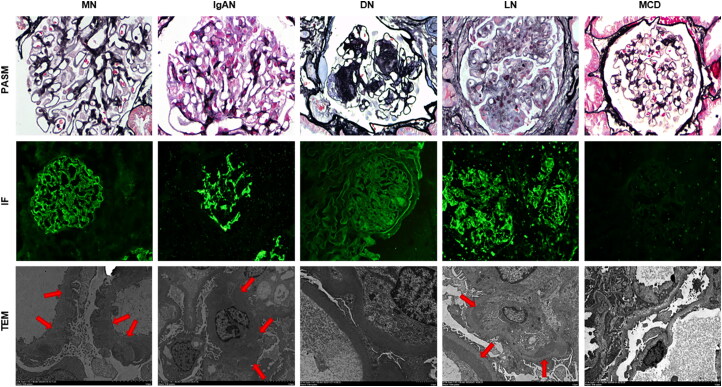
Pathological features of three kinds of renal biopsy images for different kidney diseases. In PASM-stained images with 400× magnification, MN was featured by diffuse spike formation in the basement membrane of capillaries; IgAN showed mesangial cell proliferation and stroma increase; DN exhibited nodules formation of KW and drip-like lesions in renal sacs; LN displayed global endocapillary proliferation and platinum ear formation, while MCD presented slight cell proliferation. In IF images with 200× magnification, MN revealed granular deposition of IgG and complement 3 in capillary loops, IgAN showed IgA accumulation in mesangial regions, DN exhibited linear deposition of IgG along capillary loops, and LN displayed strong positive expression of IgA, IgG, immunoglobulin M, and complement3, etc; However, the fluorescence of MCD was often negative. In electron microscope images, MN revealed subepithelial electron-dense deposits in glomeruli; IgAN showed electron-dense deposits in the mesangial region; DN exhibited no deposition of immune complex but with mesangial matrix proliferation; LN displayed electron-dense deposits in multiple locations including mesangial, subendothelial and subepithelial regions; MCD presented no electron-dense deposits, but with extensive fusion of foot processes.

To validate the comprehensive diagnostic ability of the model for MN, we analyze the renal biopsy images of 138 patients with different types of glomerular diseases, and the diseases had been diagnosed clinically (Figure 7). Among the 43 MN cases, 30 cases showing all the characteristics of typical spike formation, basement membrane granular fluorescence, and subepithelial deposits, were diagnosed as MN by the model, Additionally, 6 cases were not identified by the model as having spike formation but were still diagnosed as MN due to the detection of granular fluorescence of the basement membrane and subepithelial deposits. The remaining 7 cases showed only subepithelial deposits, with no detected spike formation or granular fluorescence of the basement membrane. Therefore, for these cases, the diagnosis of MN remains uncertain and requires further evaluation by pathologists in combination with the clinical history and serological markers, including anti-PLA2R and THSD7A antibodies (Supplementary Figure 3). For the cases of IgAN DN, and MCD, the model detected neither of spikes nor electron-dense deposits, furthermore, the fluorescence deposition manner thus ruling out MN. For LN cases, although the model detected 14 cases with spikes, all the LN cases displayed deposits in multiple regions by IF and TEM. The morphological changes suggest that they could be diagnosed as MN, which requires final confirmation by a specialist in pathology (Table 6).

**Table 6.** Comprehensive diagnosis of renal biopsy images in glomerular diseases.

	MN (43)	IgAN (40)	DN (18)	MCD (17)	LN (20)
**PASM (spike)**	30	0	0	0	14
**IF**	36 (IgG, Granular, GBM) /7(Negative)	35 (IgA, Mesangial) / 5 (Granular, GBM)	12 (IgG, Linear, GBM) / 6 (Negative)	17 (Negative)	20*
**TEM**	43 (Subepithelial deposits)	0	0	0	20*
**Diagnosis**	**36 (MN)** **/7 (suspicious)**	**No MN**	**No MN**	**No MN**	**20 (suspicious)**

*Notes:* PASM: periodic acid-silver methenamine; IF: immunofluorescence; TEM: transmission electron microscopy; MN: membranous nephropathy; IgAN: IgA nephropathy; DN: diabetic nephropathy; MCD: minimal change disease; LN: lupus nephritis; GBM: glomerular basement membrane. * indicates that its pathological characteristics are similar to those of MN and require further confirmation. Suspicious suggests that this case has some of the pathologic features of MN, but it is insufficient to diagnose MN and requires further confirmation by the pathologist in conjunction with clinical manifestations and laboratory indicators.

Summarizes the integrated diagnostic performance of our model on pathological images. The bold values highlight the results achieved in membranous nephropathy diagnosis and non-membranous nephropathy exclusion.

## Discussion

In clinical practice, the diagnosis of MN relies on a combination of clinical manifestations, laboratory tests, and renal biopsy. MN can be diagnosed in patients presenting with nephrotic syndrome and positive levels of PLA2R and THSD7A in the serum, but renal biopsy is uniquely informative in patients who are often PLA2R-negative and do not have typical nephrotic syndrome [4,30–32]. Renal biopsy is a more direct diagnostic method, which can provide more detailed and accurate information to help physicians stage and understand the lesions of renal tissues in MN to guide medication and predict prognosis. Evaluation of renal tissues is a tedious and labor-intensive task, however, the varying diagnostic levels among pathologists are inconsistent, furthermore, there is a severe shortage of pathologists, which has posed a challenge in the diagnosis and treatment of diseases that require pathological support [33,34]. Therefore, it is urgently required to develop new methods to differentiate between MN and other glomerular diseases by pathologic images.

As we know, with the development of artificial intelligence, DL has emerged as one of the most promising paradigms in clinical practice. Recent studies have highlighted the significant potential of ‘multimodal’ and ‘large model’ technologies in digital pathology [35,36]. which underscores the pressing need to integrate multimodal research methodologies into renal disease studies. DL has exhibited extensive applicability in the realm of renal diseases, including acute kidney injury, chronic kidney disease, IgAN, LN, and DN [37–41]. Nonetheless, research on MN has only recently attracted substantial attention, lagging behind other renal diseases. Notably, the majority of existing MN studies have concentrated on non-morphological methodologies [42,43]. For example, Gao et al. [42] devised a belief rule-based expert system utilizing nine biochemical indicators for the diagnosis of MN, whereas Zhang et al. [43] combined Raman spectroscopy of serum and urine samples with DL techniques for the same objective. Although DL has shown promise in MN diagnosis, most studies remain limited to high-level classification or single-modality analysis. Renal biopsy image analysis remains a relatively underexplored domain in computational pathology, hindered by three principal factors: the paucity of meticulously annotated datasets, the intricate renal architecture, and the technical complexities in multimodal data integration. To overcome these limitations, we presented DL-based frameworks specifically designed for renal morphological analysis that integrated multimodal fusion of LM, IF, and TEM images to capture key features such as subepithelial deposits, spike formations, and IgG-dominant IF patterns. This approach establishes a correlative mapping between the inferences based on images and pathological interpretation, which enhances diagnostic accuracy.

In this study, we first constructed an algorithm model based on a CNN using renal biopsy PASM-stained images, this model could detect glomerular basement membrane spikes with high precision and recall in MN patients and showed high superiority in comparative experiments with other classification network models. Wu et al. [44] established a multi-instance learning method with instance-level data augmentation to classify glomerular spikes. Chen et al. [45] proposed a glomerular classification model that uses U-Net as a data preprocessing step to segment glomeruli and ResNet to classify spike and normal glomeruli. In contrast to the previous studies, our method was based on patches for classification, which was more accurate than other classification models using whole-glomerulus images.

Considering the importance of immunocomplexes for the diagnosis of immunity-related renal diseases, IF staining was usually used to check the localizations and types of immunoglobulins. Ligabue et al. [46] explored IF images of kidneys using a CNN to determine the appearance of glomerular deposits in 2020. Pan et al. [47] proposed a new multi-task learning approach, which used the DL technique to perform quality assessment and deblurring of blurred IF images of patients to improve image clarity to facilitate accurate disease classification. These studies initially demonstrate the potential of DL techniques for application in IF image analysis. However, the performance of these reported models needs to be improved in terms of the appearance, form, and location of immune complexes. Our fluorescence image classification model was constructed using DL techniques to distinguish images with different fluorescence features. Compared with previous studies, our fluorescence classification network model is not only effective and simple at classifying different IF images of kidney diseases. Furthermore, compared with other similar models, the model presented a high accuracy, recall, and F1 score, especially for classifying fluorescence images of MN. Additionally, the model showed the capacity to accurately classify early fluorescence images in the absence of MN staging.

Electron microscopy techniques played a crucial role in renal pathology diagnosis, particularly for MN. In MN, electron-dense deposits, as key immune complex markers, were of indispensable value in the investigation and confirmation of the disease. While previous studies had concentrated on segmenting structures like podocyte-glomerular basement membrane interface, and filtration slits in renal electron microscopy images, the automated segmentation of electron-dense deposits had received limited attention [48,49]. To address this gap, we developed an innovative deposits segmentation model that specifically targets the accurate segmentation of electron-dense deposits in MN glomerular basement membranes using electron microscopy images. Experimental results demonstrated that our model exceled in segmenting electron-dense deposits, significantly reducing mis-segmentation and improving accuracy compared to existing methods. This achievement not only bridges a technological gap in the automatic segmentation of electron-dense deposits but also bolsters the accurate diagnosis of renal pathology.

To evaluate the accuracy of our system, we detected the performances of the system through three pathologists. The accuracy of our model in the classification of spikes wasnot high, and we supposed that could be due to the spikes with a subtler nature. These subtle features were often missed during visual assessments by pathologists, making it even more challenging for DL models to detect them. Therefore, incorporating additional typical images in the training sets was necessary to improve the performance of the model. Regarding fluorescence image classification, the performance of our model was superior to that of the pathologists. This was likely because the fluorescence image features of renal diseases were distinct and easily differentiated. However, the fluorescence model offered a significant advantage by dramatically reducing the time required for diagnosis. In the case of identifying electron-dense deposit segmentation, our model significantly surpassed the pathologists. The pathologists only needed to identify deposits for diagnostic purposes and thus were less rigorous in their labeling, as illustrated in Figure 5(A), whereas the model was designed to detect all deposits, which resulted in a larger discrepancy between the model and the pathologists, as well as among the pathologists themselves.

Our agreement analysis demonstrated that the model achieved performance comparable to that of expert pathologists in spike identification and IF image classification, with moderate to high Cohen’s kappa values (Figure 6(A,B)), indicating reliable detection of key diagnostic features. In contrast, the agreement among pathologists in the segmentation of electron-dense deposits was relatively limited (Figure 6(C,D)), which was probably because of the subjective nature of delineating subtle ultrastructural boundaries. Notably, the model showed comparable consistency with both pathologists and the pathologist consensus, suggesting its potential to improve the reproducibility of diagnostic assessments in renal pathology. Furthermore, our analyses of inter-observer annotation variability revealed a limited impact on the model’s segmentation performance, which indicated that the model maintained stable and reliable performance even in the presence of manual annotation variability, highlighting its robustness and potential clinical applicability. Nevertheless, further validation using larger, diverse, and multi-center datasets is warranted to confirm the generalizability of these findings.

To evaluate the generalization performance of the system, we validated it on two external test sets. As shown in Table 5, the system achieved high accuracy in classifying both PASM-stained and fluorescence images, despite variations in staining. This suggested that the model had a strong potential for external generalization. However, this approach was not optimal for the segmentation of deposits. The suboptimal performance may be attributed to differences in staining procedures between institutions, knife marks introduced during sample preparation, and variations in imaging practices. These limitations could be addressed in future studies. It is worth noting that these issues did not significantly affect the identification of deposits now that misidentifications were rare and only a few deposits were missed. Although the model in this study was validated on two independent external datasets and showed good diagnostic ability, there are still some limitations to consider. In particular, differences in sample preparation, staining methods, and imaging conditions at different research institutions may have an impact on the model’s ability to generalize.

To validate the diagnostic capability of the system for MN, images of renal biopsies from MN and other renal diseases were included in the analysis. As shown in Table 6, the model accurately identified the three lesions of MN without any misdiagnoses. In 6 of the 43 cases, although the model did not recognize the spikes, they were still diagnosed as MN by the model because of the existence of both basement membrane granular fluorescence and subepithelial deposits. This is because in the early stages of MN, the spikes have not yet been formed and only show deposition of immune complexes, and thus the model fails to recognize this feature of the spikes. In the remaining 7 cases, only subepithelial electron-dense deposits were observed, and the lack of typical spike formation and basement membrane granule fluorescence made the diagnosis of MN unclear. A combination of additional clinical information is needed to rule out other diseases and further clarify the diagnosis. For instance, PASM-stained images of LN showed spikes, but its fluorescence was strongly positive for several indicators, with deposits presented in multiple sites, and a history of systemic lupus erythematosus. The presentation of several other renal diseases differed significantly from MN, making differential diagnosis easy. Therefore, the system is worthy of further upgrade by integrating with the DL of clinical data. Preliminary testing on cases of IgAN, DN, MCD, and LN indirectly reflects the potential applicability of the model for non-MN glomerular diseases. The model demonstrated stable performance in classifying immunofluorescence images and was able to detect partial electron-dense deposits in electron microscopy. However, its accuracy on PASM-stained images was limited. This limitation highlights the MN-centric nature of the model’s initial design. Future improvements will focus on increasing the diversity of disease types in the training dataset, optimizing the model architecture, and conducting multicenter collaborative validations to enhance its generalizability and clinical utility.

In summary, this study successfully developed a multimodal DL system to diagnose MN. The glomerular spikes classification model was capable of identifying subtle glomerular lesions using PASM-stained images, and it outperformed other classification methods. The IF image classification model could accurately classify four types of fluorescence images and was superior to the pathologists. The deposit segmentation model could precisely segment subepithelial electron-dense deposits with a certain degree of stability and objectivity. The multimodal DL model could be utilized as a tool for the rapid identification of glomerular lesions in MN and serve as a reference for developing diagnostic models for other renal diseases. However, this pathology diagnostic system was only an initial study, and more images needed to be collected for future improvements. Firstly, to achieve an accurate diagnosis of other renal diseases including IgAN, DN, and LN, it is necessary to collect more images with more types of renal diseases and conduct further work. Secondly, the intention is to integrate this pathology diagnostic system with the quantitative model of renal fibrosis and the classification model of glomerular lesions, which are currently being developed, to construct a more comprehensive diagnostic and prediction platform. Third, we will explore advanced techniques, such as Transformer-based fusion, contrastive learning, and multi-view graph neural networks, to enhance model performance and clinical relevance. Finally, we will try to combine pathological images with clinical information (e.g., serological indices, urinalysis data, etc.) to enhance the clinical decision-making ability of the model, as well as expand the data sources and test the model in different hospitals and laboratory environments to evaluate its generalization ability and stability.

## Supplementary Material

Supplementary_Figure_4.tif

Supplementary_Tables.docx

Supplementary_Figure_1.tif

Supplementary_Figure_4B.tif

Supplementary_material.docx

Supplementary_Figure_4C.tif

Supplementary_Figure_3.tif

Supplementary_Figure_2.tif

Supplementary_Figure_4A.tif

## Data Availability

The data that support the findings of this study are available from the corresponding authors Pingsheng Chen (101006254@seu.edu.cn), Taotao Tang (tangtaotao90@163.com), Siyu Xia (xsy@seu.edu.cn) upon reasonable request.

## References

[1] Avasare R, Andeen N, Beck L. Novel antigens and clinical updates in membranous nephropathy. Annu Rev Med. 2024;75(1):219–332. doi: 10.1146/annurev-med-050522-034537. 37552894

[2] Hoxha E, Reinhard L, Stahl R. Membranous nephropathy: new pathogenic mechanisms and their clinical implications. Nat Rev Nephrol. 2022;18(7):466–478. doi: 10.1038/s41581-022-00564-1. 35484394

[3] Ronco P, Beck L, Debiec H, et al. Membranous nephropathy. Nat Rev Dis Primers. 2021;7(1):69. doi: 10.1038/s41572-021-00303-z. 34593809

[4] Hu X, Xu J, Wang W, et al. Combined serologic and genetic risk score and prognostication of phospholipase A2 receptor-associated membranous nephropathy. Clin J Am Soc Nephrol. 2024;19(5):573–582. doi: 10.2215/CJN.0000000000000422. 38423528 PMC11108243

[5] Luo J, Yuan Y, Tian J, et al. Clinicopathological characteristics and outcomes of PLA 2 R - associated membranous nephropathy in seropositive patients without PLA 2 R staining on kidney biopsy. Am J Kidney Dis. 2022;80(3):364–372. doi: 10.1053/j.ajkd.2022.01.426. 35288217

[6] Lu S, Xiao J, Liu D, et al. Diagnostic value of renal biopsy in anti-phospholipase A2 receptor antibody-positive patients with proteinuria in China. Sci Rep. 2024;14(1):2907. doi: 10.1038/s41598-024-53445-x. 38316889 PMC10844597

[7] Najafian B, Lusco MA, Alpers CE, et al. Approach to kidney biopsy: core curriculum 2022. Am J Kidney Dis. 2022;80(1):119–131. doi: 10.1053/j.ajkd.2021.08.024. 35125261

[8] Liang PI, Lin WC, Wen MC, et al. Learning more from the inter-rater reliability of interstitial fibrosis assessment beyond just a statistic. Sci Rep. 2023;13(1):13260. doi: 10.1038/s41598-023-40221-6. 37582967 PMC10427633

[9] Ramos J, Aung PP. International medical graduates and the shortage of US pathologists: challenges and opportunities. Arch Pathol Lab Med. 2024;148(6):735–738. doi: 10.5858/arpa.2023-0290-EP. 37787415

[10] Groh M, Badri O, Daneshjou R, et al. Deep learning-aided decision support for diagnosis of skin disease across skin tones. Nat Med. 2024;30(2):573–583. doi: 10.1038/s41591-023-02728-3. 38317019 PMC10878981

[11] Marini N, Marchesin S, Wodzinski M, et al. Multimodal representations of biomedical knowledge from limited training whole slide images and reports using deep learning. Med Image Anal. 2024;97:103303. doi: 10.1016/j.media.2024.103303. 39154617

[12] Chen S, Garcia-Uceda A, Su J, et al. Label refinement network from synthetic error augmentation for medical image segmentation. Med Image Anal. 2025;99:103355. doi: 10.1016/j.media.2024.103355. 39368280

[13] Rong Y, Lin T, Chen H, et al. Searching discriminative regions for convolutional neural networks in fundus image classification with genetic algorithms. IEEE Trans Image Process. 2024;33:5949–5958. doi: 10.1109/TIP.2024.3477932. 39412964

[14] Ziegler J, Dobsch P, Rozema M, et al. Multimodal convolutional neural network-based algorithm for real-time detection and differentiation of malignant and inflammatory biliary strictures in cholangioscopy: a proof-of-concept study (with video). Gastrointest Endosc. 2025;101(4):830.e2–842.e2. doi: 10.1016/j.gie.2024.09.001. 39265745

[15] Xu H, Usuyama N, Bagga J, et al. A whole-slide foundation model for digital pathology from real-world data. Nature. 2024;630(8015):181–188. doi: 10.1038/s41586-024-07441-w. 38778098 PMC11153137

[16] Vorontsov E, Bozkurt A, Casson A, et al. A foundation model for clinical-grade computational pathology and rare cancers detection. Nat Med. 2024;30(10):2924–2935. doi: 10.1038/s41591-024-03141-0. 39039250 PMC11485232

[17] Wang X, Zhao J, Marostica E, et al. A pathology foundation model for cancer diagnosis and prognosis prediction. Nature. 2024;634(8035):970–978. doi: 10.1038/s41586-024-07894-z. 39232164 PMC12186853

[18] Arrigo A, Aragona E, Battaglia PM, et al. Quantitative approaches in multimodal fundus imaging: state of the art and future perspectives. Prog Retin Eye Res. 2023;92:101111. doi: 10.1016/j.preteyeres.2022.101111. 35933313

[19] Gao X, Shi F, Shen D, et al. Multimodal transformer network for incomplete image generation and diagnosis of Alzheimer’s disease. Comput Med Imaging Graph. 2023;110:102303. doi: 10.1016/j.compmedimag.2023.102303. 37832503

[20] Jaltotage B, Lu J, Dwivedi G. Use of artificial intelligence including multimodal systems to improve the management of cardiovascular disease. Can J Cardiol. 2024;40(10):1804–1812. doi: 10.1016/j.cjca.2024.07.014. 39038650

[21] Parravano M, Cennamo G, Di Antonio L, et al. Multimodal imaging in diabetic retinopathy and macular edema: an update about biomarkers. Surv Ophthalmol. 2024;69(6):893–904. doi: 10.1016/j.survophthal.2024.06.006. 38942124

[22] Roest C, Yakar D, Rener SD, et al. Multimodal AI combining clinical and imaging inputs improves prostate cancer detection. Invest Radiol. 2024;59(12):854–860. doi: 10.1097/RLI.0000000000001102. 39074400

[23] Xia P, Lv Z, Wen Y, et al. Development of a multiple convolutional neural network-facilitated diagnostic screening program for immunofluorescence images of IgA nephropathy and idiopathic membranous nephropathy. Clin Kidney J. 2023;16(12):2503–2513. doi: 10.1093/ckj/sfad153. 38046020 PMC10689194

[24] Yang CK, Lee CY, Wang HS, et al. Glomerular disease classification and lesion identification by machine learning. Biomed J. 2022;45(4):675–685. doi: 10.1016/j.bj.2021.08.011. 34506971 PMC9486238

[25] Yi Z, Xi C, Menon MC, et al. A large-scale retrospective study enabled deep-learning based pathological assessment of frozen procurement kidney biopsies to predict graft loss and guide organ utilization. Kidney Int. 2024;105(2):281–292. doi: 10.1016/j.kint.2023.09.031. 37923131 PMC10892475

[26] KDIGO. Clinical practice guideline for the management of glomerular diseases. Kidney Int. 2021;100(4S):S1–S276. doi: 10.1016/j.kint.2021.05.021. 34556256

[27] Bajema IM, Wilhelmus S, Alpers CE, et al. Revision of the International Society of Nephrology/Renal Pathology Society classification for lupus nephritis: clarification of definitions, and modified National Institutes of Health activity and chronicity indices. Kidney Int. 2018;93(4):789–796. doi: 10.1016/j.kint.2017.11.023. 29459092

[28] He K, Zhang X, Ren S, et al. Deep residual learning for image recognition. In: 2016 IEEE Conference on Computer Vision and Pattern Recognition (CVPR). Las Vegas, NV, USA; 2016. p. 770–778. doi: 10.1109/CVPR.2016.90.

[29] Selvaraju RR, Cogswell M, Das A, et al. Grad-CAM: visual explanations from deep networks via gradient-based localization. Int J Comput Vis. 2020;128(2):336–359. doi: 10.1007/s11263-019-01228-7.

[30] Bobart SA, Han H, Tehranian S, et al. Noninvasive diagnosis of PLA2R-associated membranous nephropathy: a validation study. Clin J Am Soc Nephrol. 2021;16(12):1833–1839. doi: 10.2215/CJN.05480421. 34782349 PMC8729491

[31] Maifata SM, Hod R, Zakaria F, et al. Role of serum and urine biomarkers (PLA 2 R and THSD7A) in diagnosis, monitoring and prognostication of primary membranous glomerulonephritis (retracted article). Biomolecules. 2020;10(2):319. doi: 10.3390/biom10020319. 32079308 PMC7072431

[32] Roccatello D, Fenoglio R, Sciascia S. The role of kidney biopsy in the diagnosis of membranous nephropathy. Clin Kidney J. 2024;17(10):sfae292. doi: 10.1093/ckj/sfae292. 39435319 PMC11491826

[33] D’Abbronzo G, Lucà S, Carraturo E, et al. Shortage of pathologists in Italy: survey of students and residents. Pathologica. 2023;115(3):172–180. doi: 10.32074/1591-951X-852. 37387442 PMC10462991

[34] Dasari S, Chakraborty A, Truong L, et al. A systematic review of interpathologist agreement in histologic classification of lupus nephritis. Kidney Int Rep. 2019;4(10):1420–1425. doi: 10.1016/j.ekir.2019.06.011. 31701051 PMC6829183

[35] Chen RJ, Ding T, Lu MY, et al. Towards a general-purpose foundation model for computational pathology. Nat Med. 2024;30(3):850–862. doi: 10.1038/s41591-024-02857-3. 38504018 PMC11403354

[36] Li Y, El HDM, Conze PH, et al. A review of deep learning-based information fusion techniques for multimodal medical image classification. Comput Biol Med. 2024;177:108635. doi: 10.1016/j.compbiomed.2024.108635. 38796881

[37] Lei Q, Hou X, Liu X, et al. Artificial intelligence assists identification and pathologic classification of glomerular lesions in patients with diabetic nephropathy. J Transl Med. 2024;22(1):397. doi: 10.1186/s12967-024-05221-8. 38684996 PMC11059590

[38] Shi M, Chen C, Liu L, et al. A grade-based search adaptive random slime mould optimizer for lupus nephritis image segmentation. Comput Biol Med. 2023;160:106950. doi: 10.1016/j.compbiomed.2023.106950. 37120988

[39] Testa F, Fontana F, Pollastri F, et al. Automated prediction of kidney failure in iga nephropathy with deep learning from biopsy images. Clin J Am Soc Nephrol. 2022;17(9):1316–1324. doi: 10.2215/CJN.01760222. 35882505 PMC9625090

[40] Wu C, Zhang Y, Nie S, et al. Predicting in-hospital outcomes of patients with acute kidney injury. Nat Commun. 2023;14(1):3739. doi: 10.1038/s41467-023-39474-6. 37349292 PMC10287760

[41] Zhao X, Gu X, Meng L, et al. Screening chronic kidney disease through deep learning utilizing ultra-wide-field fundus images. NPJ Digit Med. 2024;7;7(1):275. doi: 10.1038/s41746-024-01271-w. PMC11458603 39375513

[42] Gao J, Wang S, Xu L, et al. Computer-aided diagnosis of primary membranous nephropathy using expert system. Biomed Eng Online. 2023;22(1):6. doi: 10.1186/s12938-023-01063-5. 36732817 PMC9893592

[43] Zhang X, Song X, Li W, et al. Rapid diagnosis of membranous nephropathy based on serum and urine Raman spectroscopy combined with deep learning methods. Sci Rep. 2023;13(1):3418. doi: 10.1038/s41598-022-22204-1. 36854769 PMC9974944

[44] Wu X, Chen Y, Li X, et al. IDA-MIL: classification of glomerular with spike-like projections via multiple instance learning with instance-level data augmentation. Comput Methods Programs Biomed. 2022;225:107106. doi: 10.1016/j.cmpb.2022.107106. 36088891

[45] Chen Y, Li M, Hao F, et al., editors. Classification of glomerular spikes using convolutional neural network. Taiyuan, China: Association for Computing Machinery; 2020.

[46] Ligabue G, Pollastri F, Fontana F, et al. Evaluation of the classification accuracy of the kidney biopsy direct immunofluorescence through convolutional neural networks. Clin J Am Soc Nephrol. 2020;15(10):1445–1454. doi: 10.2215/CJN.03210320. 32938617 PMC7536749

[47] Pan S, Fu Y, Chen P, et al. Multi-task learning-based immunofluorescence classification of kidney disease. Int J Env Res Public Health. 2021;18(20):10798. doi: 10.3390/ijerph182010798. PMC8535636 34682567

[48] Smerkous D, Mauer M, Tøndel C, et al. Development of an automated estimation of foot process width using deep learning in kidney biopsies from patients with Fabry, minimal change, and diabetic kidney diseases. Kidney Int. 2024;105(1):165–176. doi: 10.1016/j.kint.2023.09.011. 37774924 PMC10842003

[49] Unnersjö-Jess D, Butt L, Höhne M, et al. Deep learning-based segmentation and quantification of podocyte foot process morphology suggests differential patterns of foot process effacement across kidney pathologies. Kidney Int. 2023;103(6):1120–1130. doi: 10.1016/j.kint.2023.03.013. 36990215

