# Supplementary figures and images for: Multimodal deep learning improving the accuracy of pathological diagnoses for membranous nephropathy
Source: Ren Fail. 2025 Jul 14;47(1):2528106. doi: 10.1080/0886022X.2025.2528106 (PMC12261511; doi:10.1080/0886022X.2025.2528106)

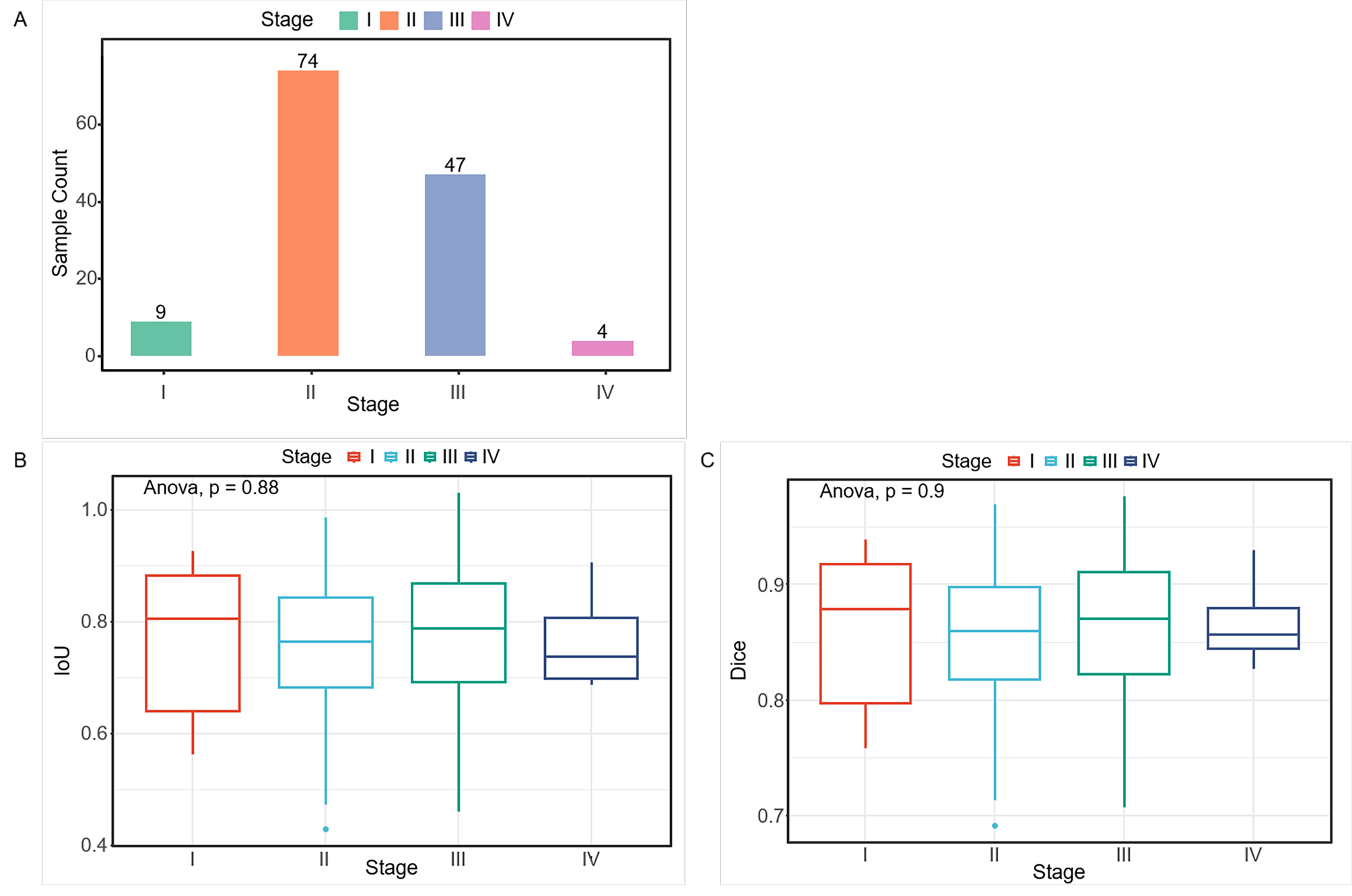

Supplement: Supplementary_Figure_4.tif [file IRNF_A_2528106_SM0463.tif]

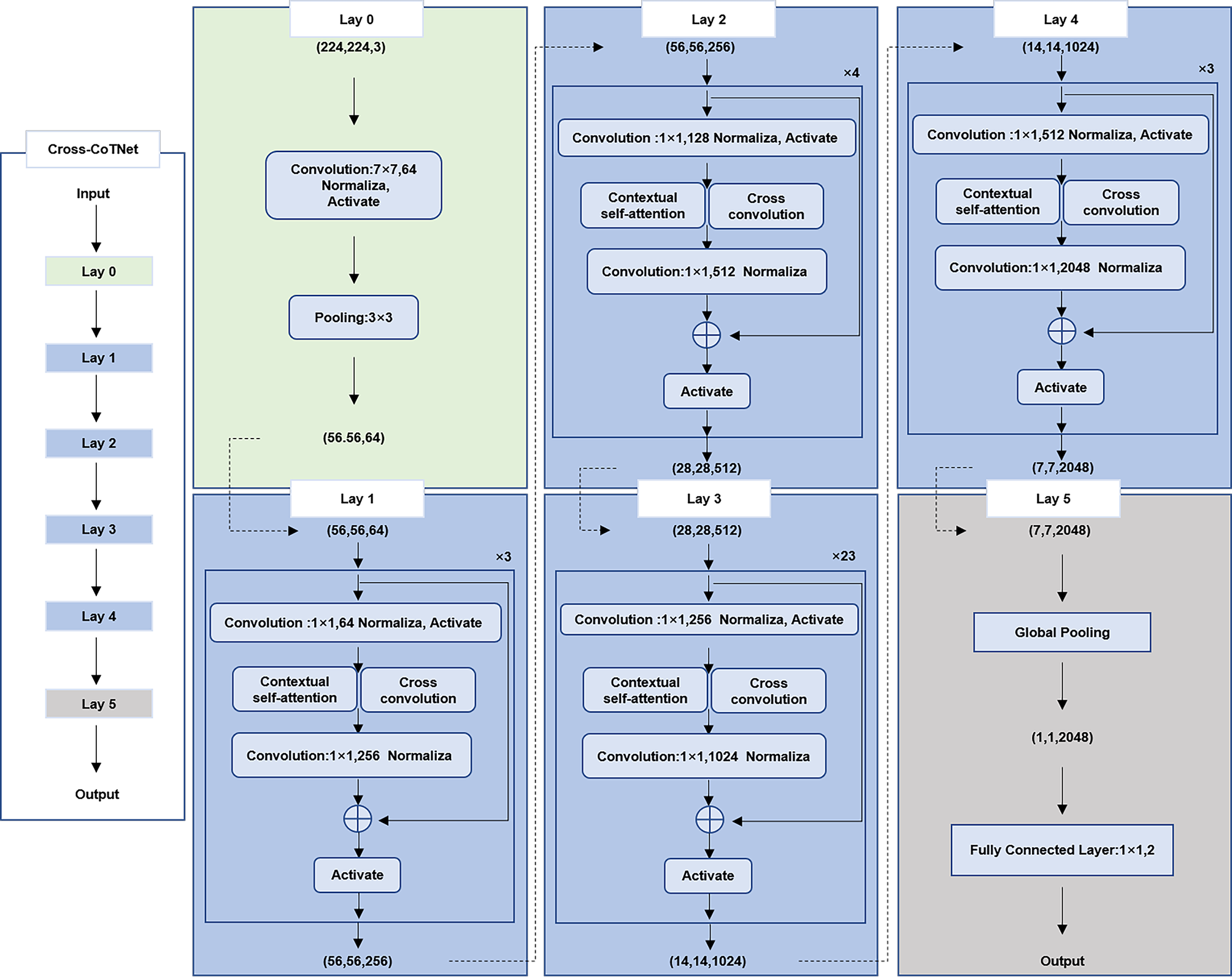

Supplement: Supplementary_Figure_1.tif [file IRNF_A_2528106_SM0461.tif]

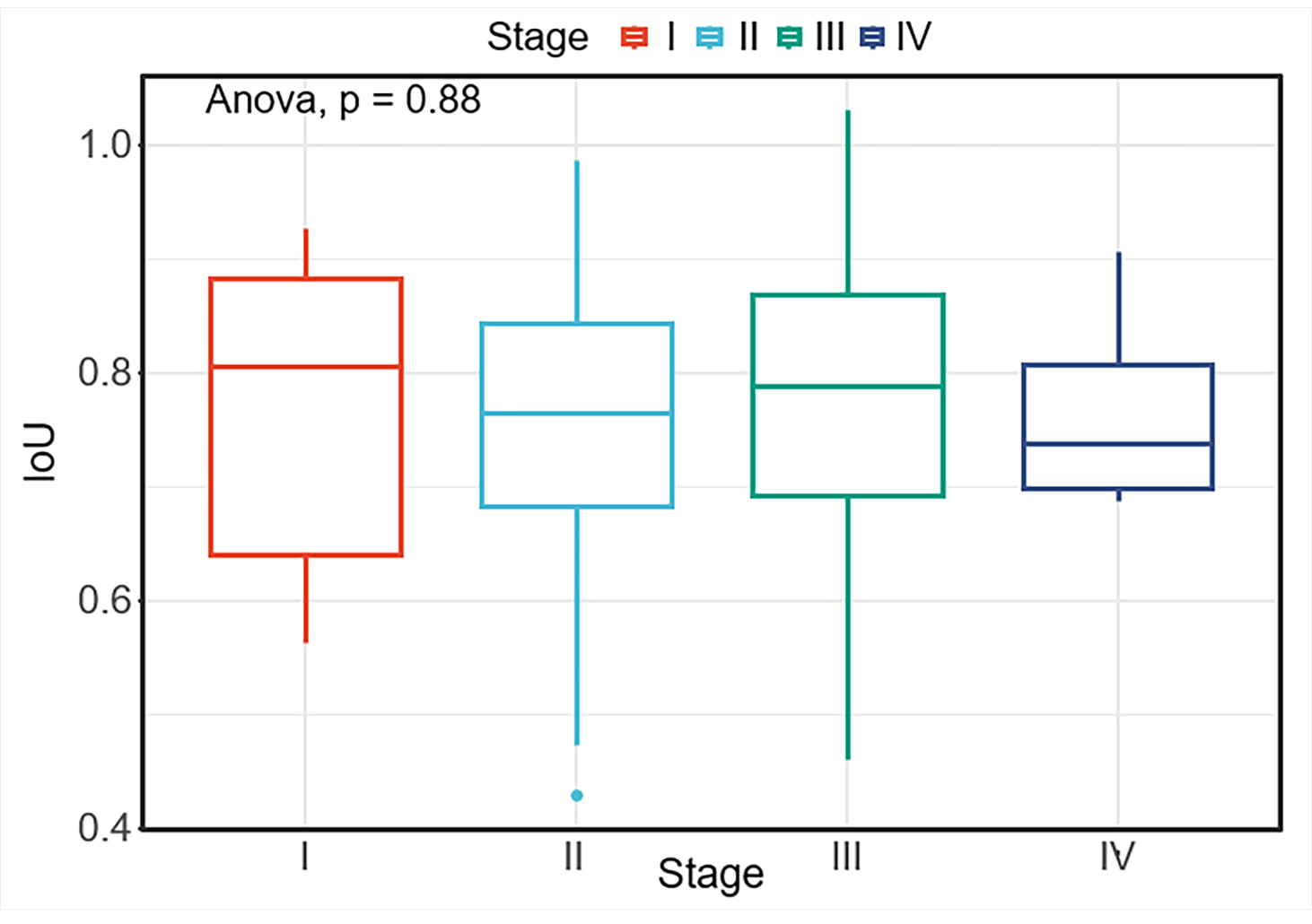

Supplement: Supplementary_Figure_4B.tif [file IRNF_A_2528106_SM0460.tif]

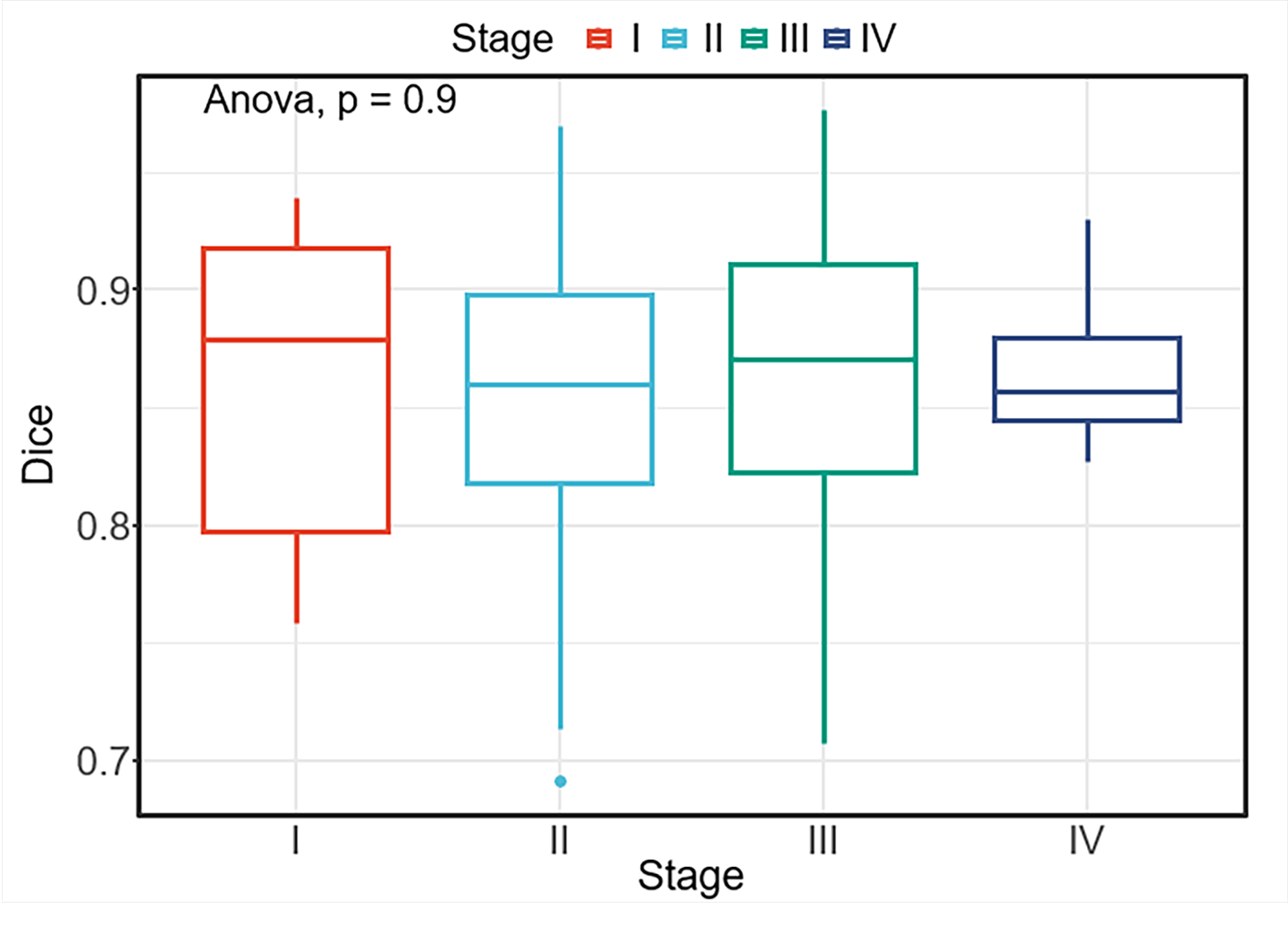

Supplement: Supplementary_Figure_4C.tif [file IRNF_A_2528106_SM0458.tif]

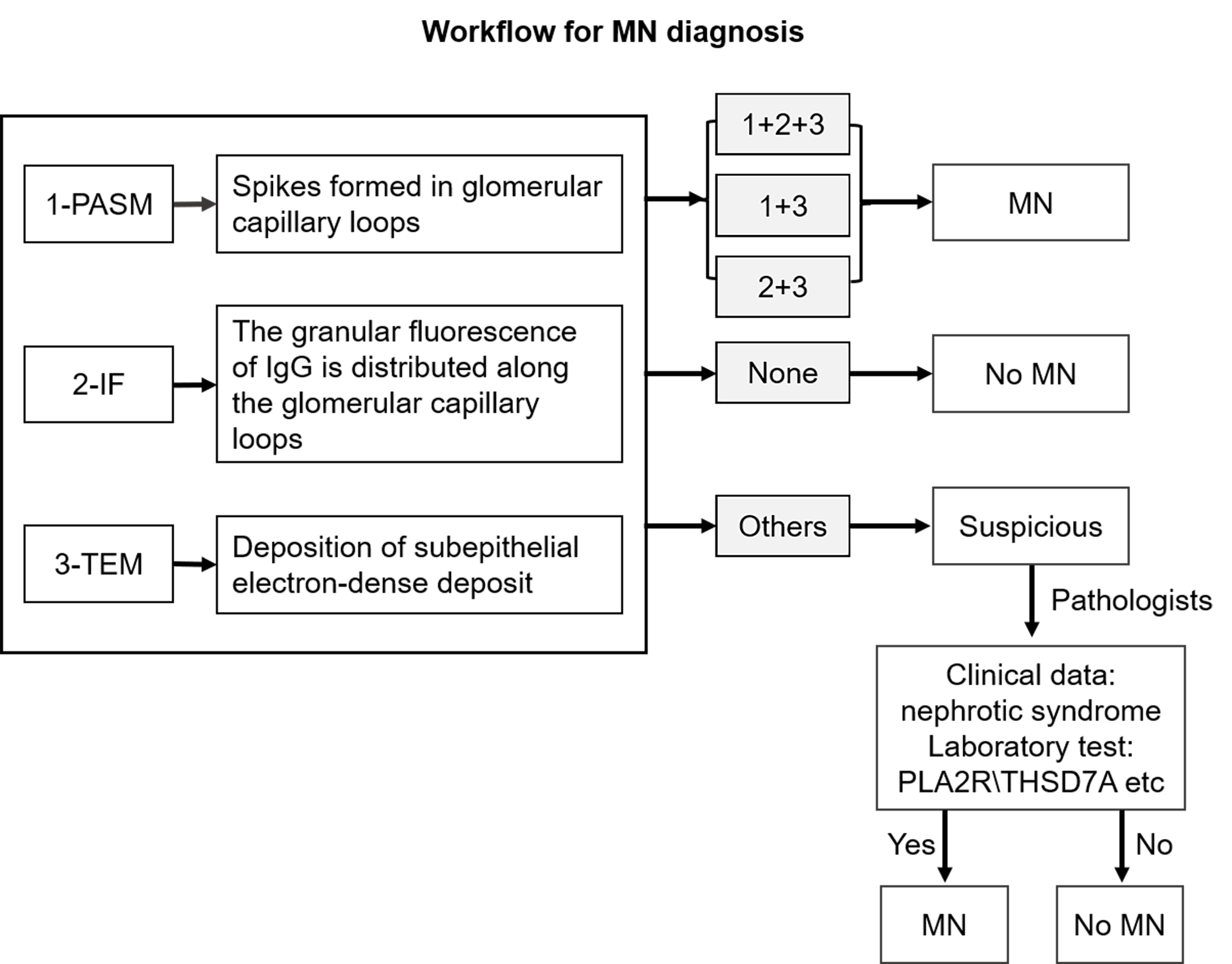

Supplement: Supplementary_Figure_3.tif [file IRNF_A_2528106_SM0457.tif]

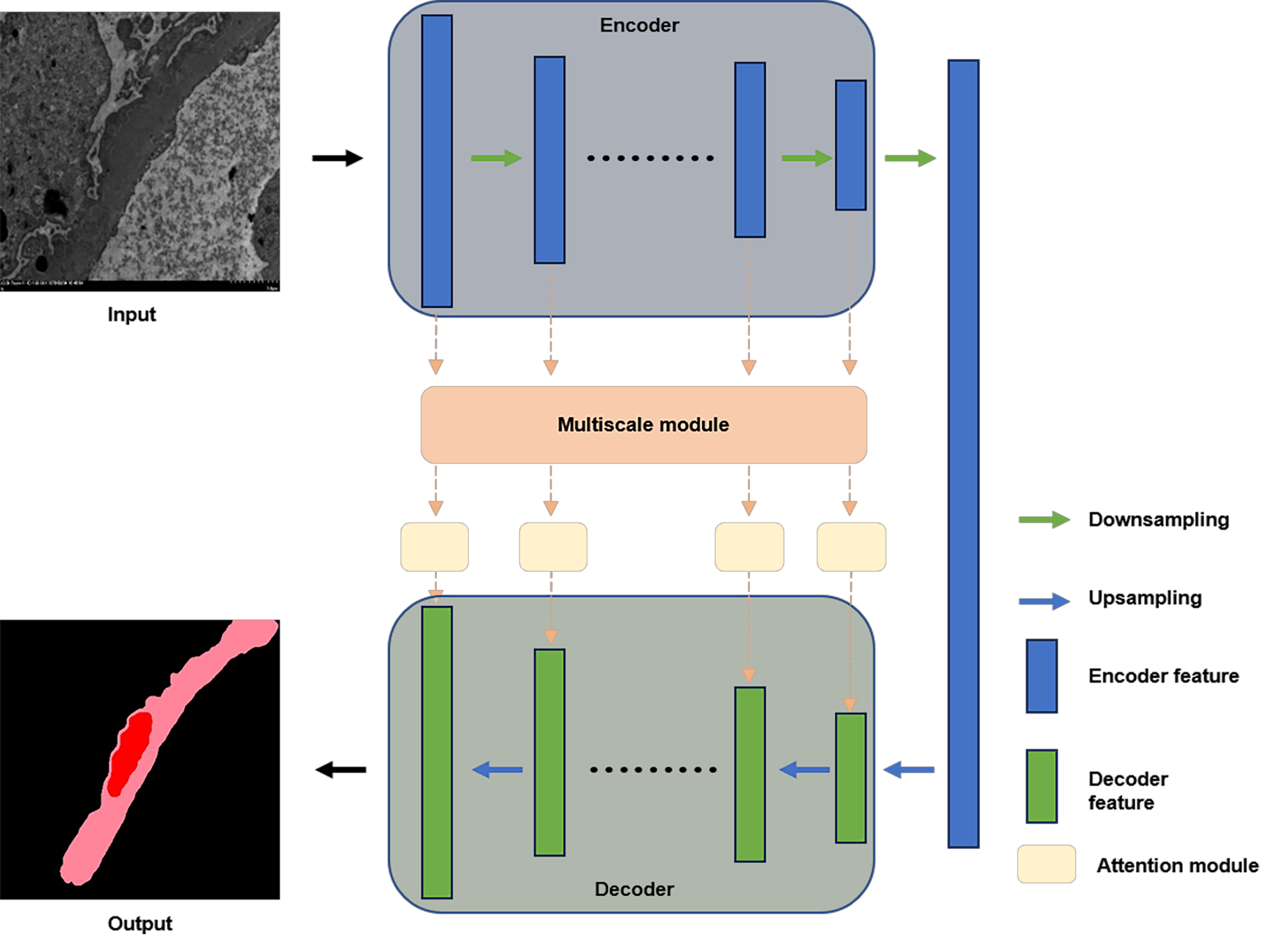

Supplement: Supplementary_Figure_2.tif [file IRNF_A_2528106_SM0456.tif]

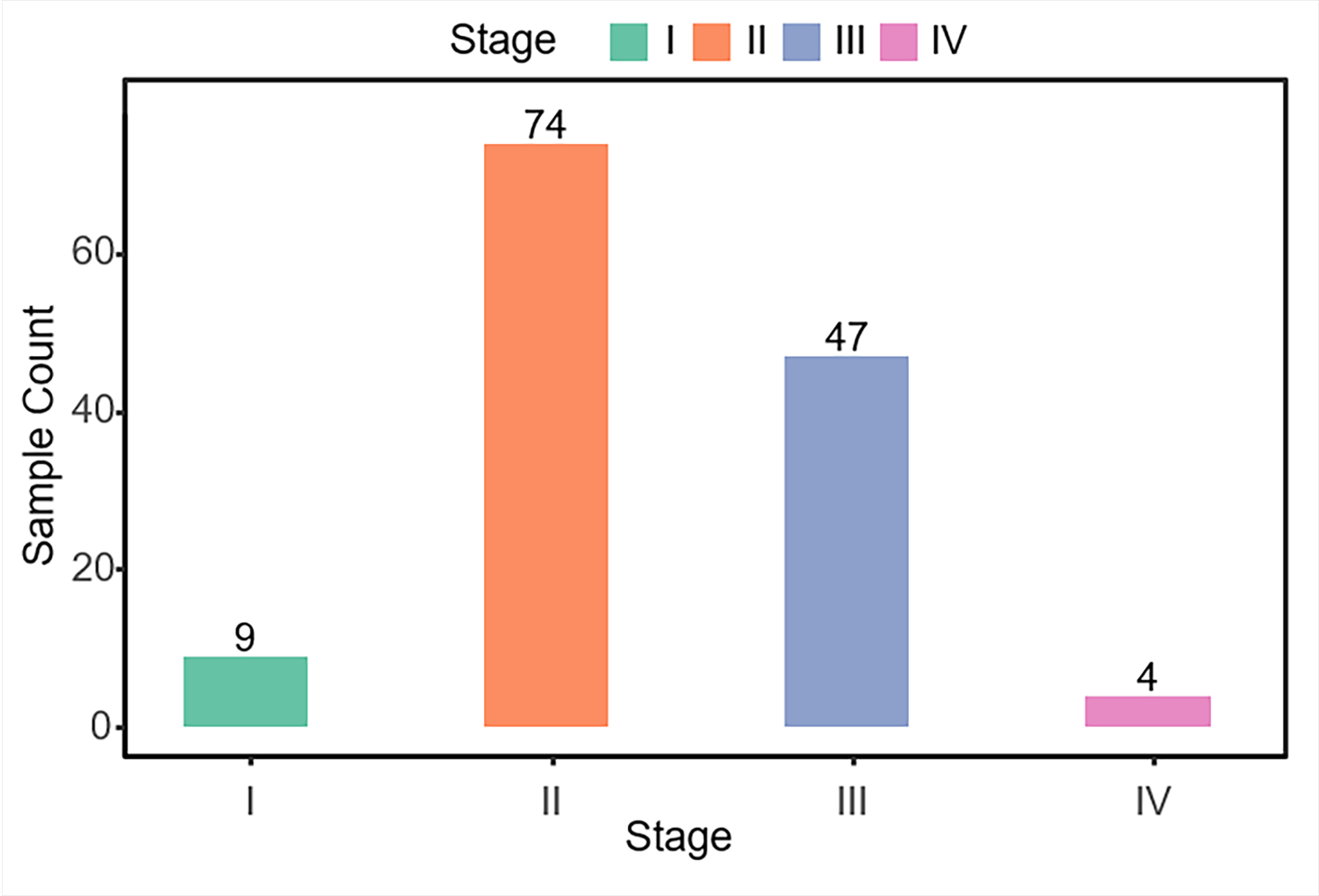

Supplement: Supplementary_Figure_4A.tif [file IRNF_A_2528106_SM0455.tif]
