# Supplementary material for: Multimodal deep learning improving the accuracy of pathological diagnoses for membranous nephropathy
Source: Ren Fail. 2025 Jul 14;47(1):2528106. doi: 10.1080/0886022X.2025.2528106 (PMC12261511; doi:10.1080/0886022X.2025.2528106)
Supplement: Supplementary_Tables.docx [file IRNF_A_2528106_SM0462.docx]

**Supplementary Table 1.** Network structure of fluorescent classification.

| **Layer** | **Resnext-101 (**$\boldsymbol{32\times4d)}$ | **D-resnext (**$\boldsymbol{32\times32d)}$ |
| --- | --- | --- |
| 0 | $7\times7, output channel number64, stride2$ | $7\times7, output channel number64, stride2$ |
| 1 | $3\times3, \text{max pooling}, stride2$ | $3\times3\text{, }\text{max pooling}, stride2$ |
|  | $3\times\left\{ \begin{aligned} 1\times1, output channel number128 \\ 3\times3, output channel number128 \\ 1\times1, output channel number256 \end{aligned} \right.$ | $3\times\left\{ \begin{aligned} 1\times1, output channel number1024 \\ 3\times3, output channel number1024 \\ 1\times1, output channel number256 \end{aligned} \right.$ |
| 2 | $4\times\left\{ \begin{aligned} 1\times1, output channel number256 \\ 3\times3, output channel number256 \\ 1\times1, output channel number512 \end{aligned} \right.$ | $4\times\left\{ \begin{aligned} 1\times1, output channel number2048 \\ 3\times3, output channel number2048 \\ 1\times1, output channel number512 \end{aligned} \right.$ |
| 3 | $23\times\left\{ \begin{aligned} 1\times1, output channel number512 \\ 3\times3, output channel number512 \\ 1\times1, output channel number1024 \end{aligned} \right.$ | $23\times\left\{ \begin{aligned} 1\times1, output channel number4096 \\ 3\times3, output channel number4096 \\ 1\times1, output channel number1024 \end{aligned} \right.$ |
| 4 | $3\times\left\{ \begin{aligned} 1\times1, output channel number1024 \\ 3\times3, output channel number1024 \\ 1\times1, output channel number2048 \end{aligned} \right.$ | $3\times\left\{ \begin{aligned} 1\times1, output channel number8192 \\ 3\times3, output channel number8192 \\ 1\times1, output channel number512 \end{aligned} \right.$ |
| 5 | Global pooling, Fully connected layer, Activation function | |

ResNeXt includes a base in addition to the depth and width of the network and uses multibranch convolution to improve the fitting ability, which improves its ability to represent network features. However, the inputs of the middle ResNeXt layer were related to only the outputs of the previous layer and did not fully exploit the features already computed by the upper layer. Therefore, in this study, we added dense connection to the ResNeXt network so that the inputs of some of the middle network layers were the output features of the previous layers and constructed a model that can classify glomerular immunofluorescence images.

The network has a total of 101 layers, of which only the convolutional and fully connected layers are computed; pooling, normalization, and activation functions are not computed. In the last three convolutional blocks, dense multi-branch convolution is used in this study. The first convolutional layer is used to partition the input features into low-dimensional features by reducing the number of channels of the feature map to 32 by 1× 1 convolution, the second convolutional layer is a conventional 3 × 3 convolution used for feature extraction, and the third convolution is used to aggregate the low-dimensional features from different branches to convert the number of channels of the features to the same dimensions as the input. The final output of the module consists of the output of the third convolutional layer and the input feature map of the first convolutional layer, spliced together to realize a dense concatenation.

**Supplementary Table 2.** Training configuration.

| **Model** | | **Parameter** | **Value** |
| --- | --- | --- | --- |
| Classification model for spikes | Backbone Network | | ResNet |
|  | Optimizer | | SGD |
|  | Initial learning rate | | 0.5 |
|  | Batch size | | 20 |
|  | Epochs | | 160 |
|  | Momentum | | 0.9 |
|  | Weight decay | | 0.0005 |
|  | Input image size | | 224 × 224 |
|  | Number of classes | | 2 (positive / negative) |
|  | Hardware | | NVIDIA GTX 2080Ti GPU |
| Classification model for fluorescence images | Backbone Network | | ResNeXt |
|  | Optimizer | | SGD |
|  | Initial learning rate | | 0.001 |
|  | Batch size | | 4 |
|  | Epochs | | 50 |
|  | Momentum | | 0.9 |
|  | Weight decay | | 0.0005 |
|  | Loss function | | Cross Entropy |
|  | Input image size | | 1024 × 1024 |
|  | Number of classes | | 4(MN/IgAN/DN/Negative) |
|  | Hardware | | NVIDIA GTX 2080Ti GPU |
| Segmentation model for electron-dense deposits | Backbone Network | | U-Net |
|  | Optimizer | | Adam |
|  | Initial learning rate | | 0.01 |
|  | Batch size | | 2 |
|  | Epochs | | 150 |
|  | Momentum | | 0.99 |
|  | Weight decay | | 0.00003 |
|  | Loss function | | Cross Entropy + Dice loss |
|  | Input image size | | 512 × 512 |
|  | Hardware | | NVIDIA GTX 2080Ti GPU |

**Supplementary Table 3.** Confusion matrix.

| Ground Truth | Prediction | |
| --- | --- | --- |
|  | Positive | Negative |
| Positive | TP | FN |
| Negative | FP | TN |

In particular, for TP (true positives): the classifier predicts a positive result that is actually positive; that is, the number of positive samples correctly identified. FP (false positive): the classifier predicts positive samples that are actually negative; that is, the number of false negative samples. TN (true negative): The classifier predicts a negative sample that is actually negative; that is, the number of negative samples that are correctly identified. FN (false negative): the classifier predicts negative samples that are actually positive; that is, the number of positive samples that are underreported.

**Supplementary Table 4.** Results of spike classification ablation experiment.

| **Module** | | **Precision (%)** | **Recall (%)** | **F1(%)** |
| --- | --- | --- | --- | --- |
| **Contextual self-attention** | **Cross-convolution** |  |  |  |
| - | + | 90.2 | 75.41 | 82.14 |
| + | - | 91.59 | 80.33 | 85.59 |
| **+** | **+** | **91.74** | **81.97** | **86.58** |

From the data in the table, it can be seen that the network with contextual self-attention improves precision, recall, and F1 by 1.54%, 6.56%, and 4.44%, respectively, compared to the model improved by adding cross-convolution only. Similarly, the addition of cross-convolution improves precision, recall, and F1 by 0.15%, 1.64%, and 0.99%, respectively, compared to the model improved by adding contextual self-attention only. However, the improvement resulting from the addition of cross-convolution is less pronounced than that resulting from the addition of contextual self-attention, indicating that contextual self-attention plays a significant role in improving the model's classification performance.

**Supplementary Table 5.** Results of electron dense ablation experiment.

| **Methods** | | **Dice (%)** | **IOU (%)** |
| --- | --- | --- | --- |
| Multiscale | Attention |  |  |
| Baseline | | 80.5 | 69.1 |
| + | - | 82.8 | 71.8 |
| - | + | 81.7 | 70.5 |
| **+** | **+** | **85.6** | **75.9** |

As shown in the table, the Dice coefficient and IOU coefficient increased by 2.3% and 2.7%, respectively, after the addition of multiscale connectivity, and the use of channel attention alone increased the Dice coefficient and IOU coefficient of the baseline model by 1.2% and 1.4%, respectively. The simultaneous application of multiscale connectivity and channel attention increased the Dice and IOU coefficients of the baseline model by 5.1% and 6.8%, separately. This implies that both multiscale connectivity and channel attention contribute to the segmentation performance of the network model, and the combination of multiscale connectivity and channel attention significantly improves the segmentation accuracy.
