# Supplementary material for: Multimodal deep learning improving the accuracy of pathological diagnoses for membranous nephropathy
Source: Ren Fail. 2025 Jul 14;47(1):2528106. doi: 10.1080/0886022X.2025.2528106 (PMC12261511; doi:10.1080/0886022X.2025.2528106)
Supplement: Supplementary_material.docx [file IRNF_A_2528106_SM0459.docx]

**SUPPLEMENTARY MATERIAL**

Supplementary Figure 1. The network structure of spike classification.

Supplementary Figure 2. The network structure of electron-dense deposit segmentation.

Supplementary Figure 3.Clinical workflow integrating AI model output with pathological and serological data for MN diagnosis.

Supplementary Figure 4. Distribution of MN stages and corresponding model segmentation performance.

Supplementary Table 1. The network structure of the fluorescent classification.

Supplementary Table 2. Training configuration.

Supplementary Table 3. Confusion matrix.

Supplementary Table 4. Results of spike classification ablation experiment.

Supplementary Table 5. Results of electron-dense ablation experiment.

Supplementary Methods 1: Model formula.

Supplementary Methods 2: Stage-wise performance and annotation variability analysis.

Supplementary Result: Model generalization across MN stages and resistance to annotation inconsistency.


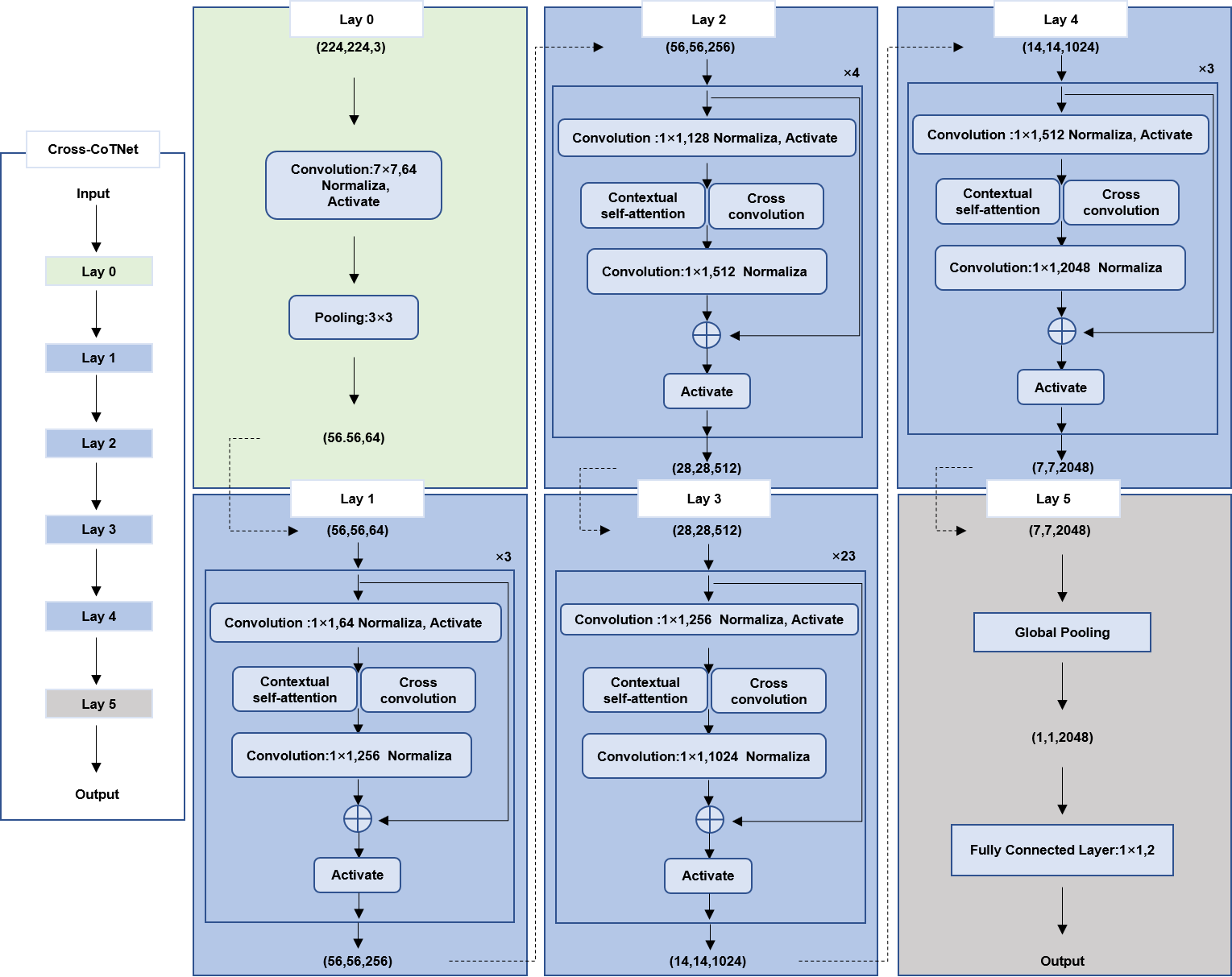


**Supplementary Figure 1.** Network structure of spike classification.

According to the PASM-stained images in this study, the spikes were relatively small, the neighboring capillary loops tended to have different degrees of lesions, and the capillary distribution was relatively cohesive; all of the capillaries were within the glomerulus, so information from the immediate neighbors was more important for feature extraction. Contextual self-attention was incorporated into the backbone network to emphasize the characteristics of proximate tissues, and the data between adjacent capillary collaterals were analyzed to improve lesion detection. In addition, because glomerular basement membrane spike lesions are mostly focused on the epithelial side of capillary loops according to PASM-stained, extracting image edge information features is crucial for accurate sample classification. The model was enhanced with cross-convolution to preserve edge information to increase the attention of the network to the spike, which in turn helps the attention module to enhance edge features, leading to better spike recognition.

Cross-CoTNet adopts the residual network as the framework backbone network, and divides the network into six layers based on the number of channels of the feature map. The network has 101 convolutional layers, the first 100 layers are used to extract the feature information, and the last layer uses the fully connected layer and activation function to classify the feature information. The contextual self-attention module will enhance the self-attention by exploiting the rich context between the input keys of the feature map to focus on the neighboring features. The cross-convolution consists of two decomposed asymmetric filters, the application of two asymmetric filters increases the matrix rank, retains more structural information, and emphasizes the edge information by utilizing both vertical and horizontal gradient information.


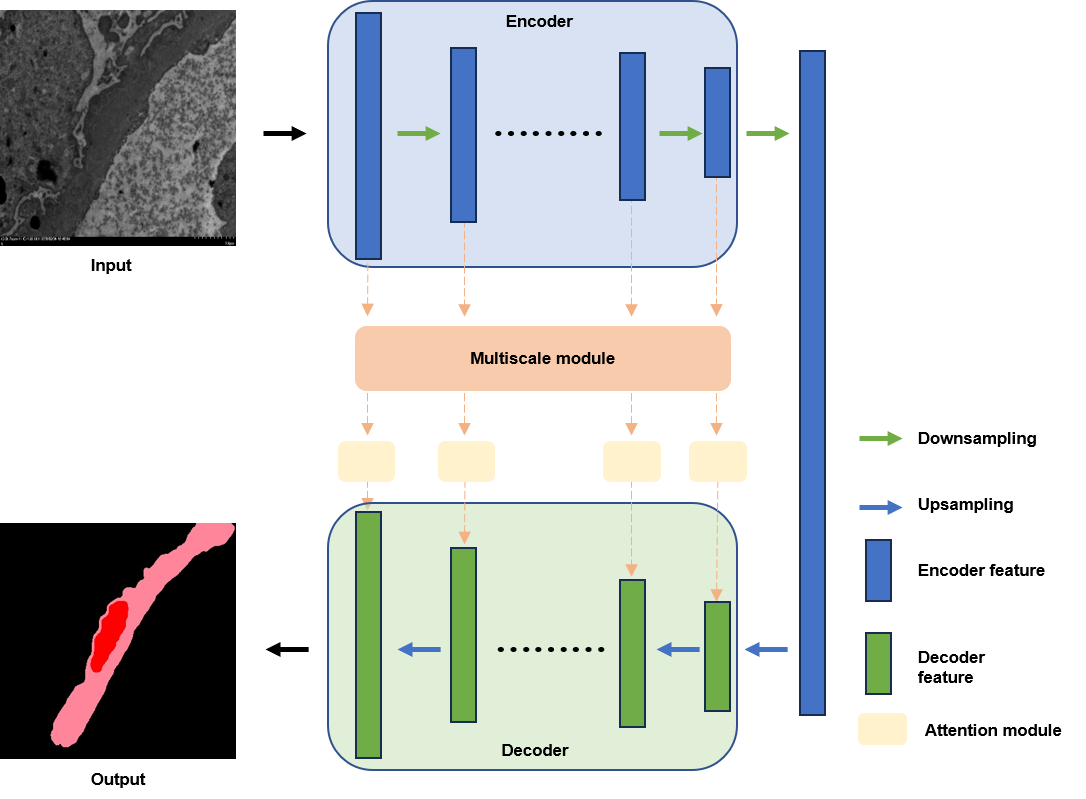


**Supplementary Figure 2.** Network structure of electron-dense deposits segmentation.

AGMS-UNet uses a U-shaped encoder-decoder network as the framework backbone, with the addition of multiscale modules and skip connected attention modules. The left half of the U-network is the encoder path. In the encoder path, the input image is downsampled through a convolutional layer and a pooling layer to obtain a feature map. The right half of the U-network is the decoder path. The multiscale module is optimized for skip connection, and the feature maps of different layers of the coding path are fused in the decoding path to reduce the loss of feature information. The attention mechanism selectively emphasizes informative features and suppresses redundant features.


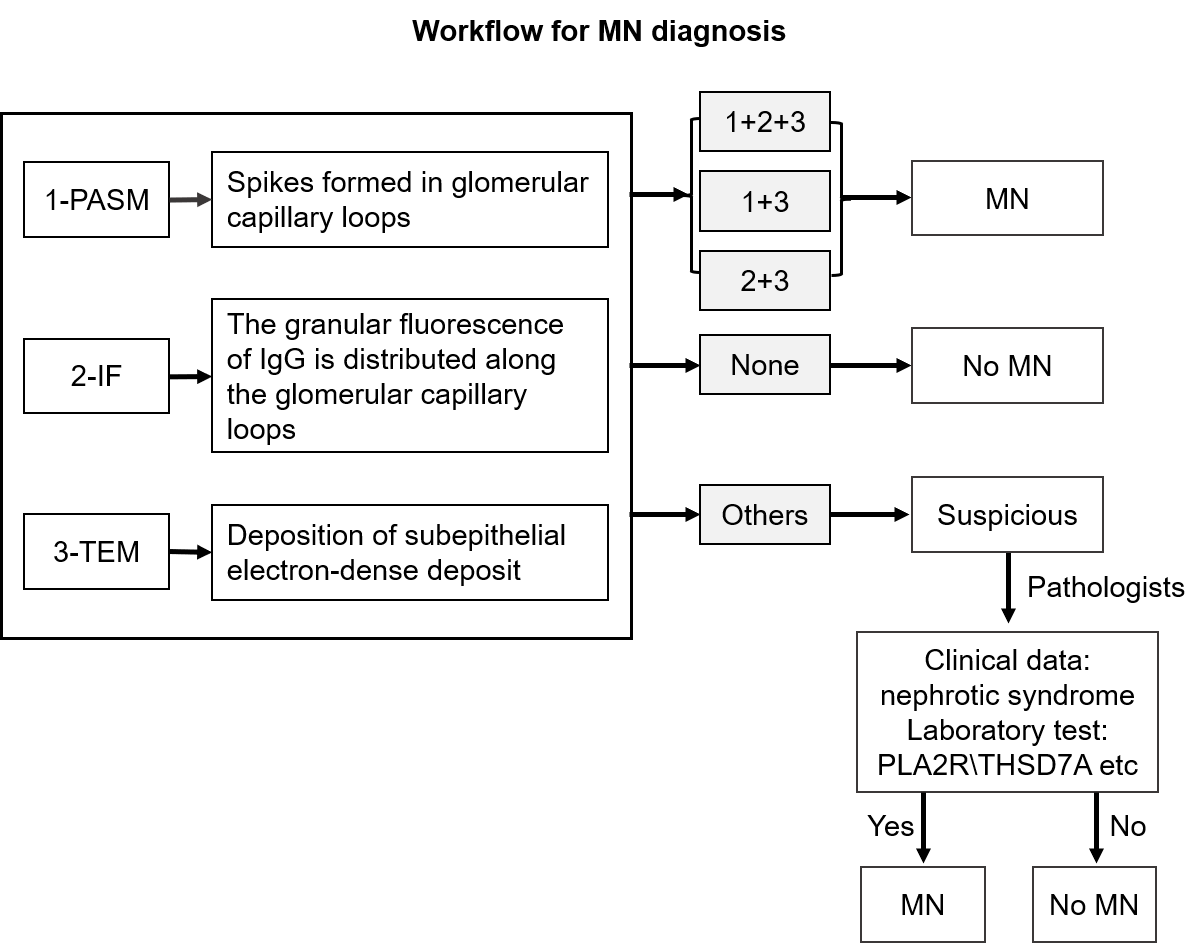


**Supplementary Figure 3. Clinical workflow integrating AI model output with pathological and serological data for MN diagnosis.**

This diagram illustrates the real-world diagnostic workflow for MN incorporating the AI model. Initial assessment is performed using pathological images (IF, LM, EM). When essential features (e.g., subepithelial spikes, immune deposits) are confidently detected, the model issues a “positive” prediction. In the cases lacking definitive features, the model assigns an “uncertainty score” and classifies the case as “suspicious.” These cases are subsequently reviewed by pathologists who integrate additional clinical and serological data（anti-PLA2R and THSD7A antibody，etc）—before making the final diagnosis. This workflow underscores the role of the model as an assistive tool to flag ambiguous cases for further clinical scrutiny.


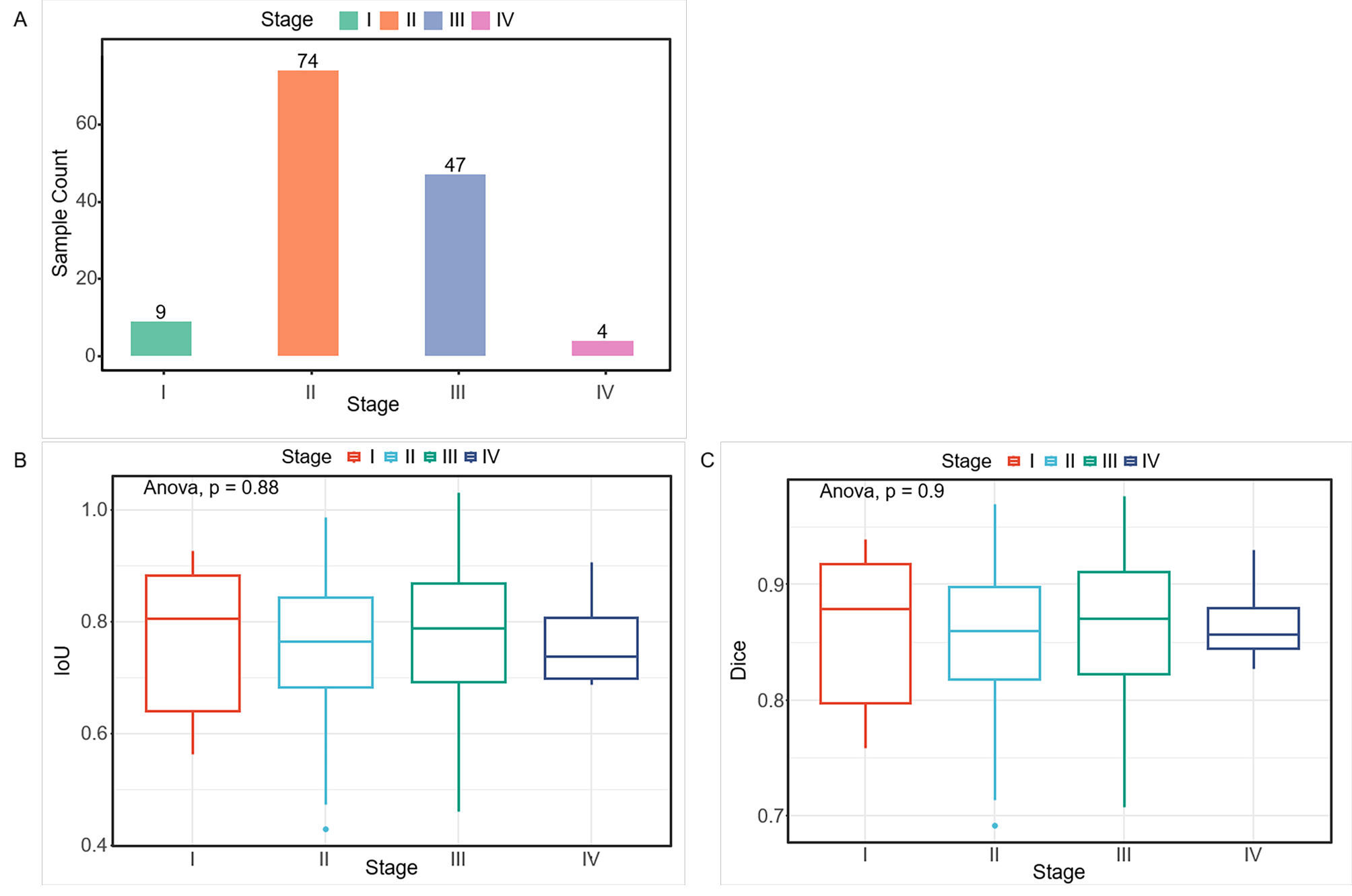


**Supplementary Figure 4. Distribution of MN stages and corresponding model segmentation performance.**

(A) Distribution of MN stages in the test set: The histogram illustrating the distribution of 134 TEM images across MN pathological stages I–IV. Stage II represented the largest proportion of cases, followed by stages III, I, and IV.

(B) Stage-IoU scores: The box plot displaying the model’s IoU scores across different MN stages. No statistical significance was detected among different stages (ANOVA, p = 0.88), indicating stable segmentation performance throughout disease progression.

(C) Stage-Dice scores: The box plot illustrating the model’s Dice similarity coefficients scores through various MN stages. No significant differences were examined (ANOVA, p = 0.90), supporting the model’s robustness across various stages of disease.

**Supplementary Table 1.** Network structure of fluorescent classification.

| **Layer** | **Resnext-101 (**$\boldsymbol{32\times4d)}$ | **D-resnext (**$\boldsymbol{32\times32d)}$ |
| --- | --- | --- |
| 0 | $7\times7, output channel number64, stride2$ | $7\times7, output channel number64, stride2$ |
| 1 | $3\times3, \text{max pooling}, stride2$ | $3\times3\text{, }\text{max pooling}, stride2$ |
|  | $3\times\left\{ \begin{aligned} 1\times1, output channel number128 \\ 3\times3, output channel number128 \\ 1\times1, output channel number256 \end{aligned} \right.$ | $3\times\left\{ \begin{aligned} 1\times1, output channel number1024 \\ 3\times3, output channel number1024 \\ 1\times1, output channel number256 \end{aligned} \right.$ |
| 2 | $4\times\left\{ \begin{aligned} 1\times1, output channel number256 \\ 3\times3, output channel number256 \\ 1\times1, output channel number512 \end{aligned} \right.$ | $4\times\left\{ \begin{aligned} 1\times1, output channel number2048 \\ 3\times3, output channel number2048 \\ 1\times1, output channel number512 \end{aligned} \right.$ |
| 3 | $23\times\left\{ \begin{aligned} 1\times1, output channel number512 \\ 3\times3, output channel number512 \\ 1\times1, output channel number1024 \end{aligned} \right.$ | $23\times\left\{ \begin{aligned} 1\times1, output channel number4096 \\ 3\times3, output channel number4096 \\ 1\times1, output channel number1024 \end{aligned} \right.$ |
| 4 | $3\times\left\{ \begin{aligned} 1\times1, output channel number1024 \\ 3\times3, output channel number1024 \\ 1\times1, output channel number2048 \end{aligned} \right.$ | $3\times\left\{ \begin{aligned} 1\times1, output channel number8192 \\ 3\times3, output channel number8192 \\ 1\times1, output channel number512 \end{aligned} \right.$ |
| 5 | Global pooling, Fully connected layer, Activation function | |

ResNeXt includes a base in addition to the depth and width of the network and uses multibranch convolution to improve the fitting ability, which improves its ability to represent network features. However, the inputs of the middle ResNeXt layer were related to only the outputs of the previous layer and did not fully exploit the features already computed by the upper layer. Therefore, in this study, we added dense connection to the ResNeXt network so that the inputs of some of the middle network layers were the output features of the previous layers and constructed a model that can classify glomerular immunofluorescence images.

The network has a total of 101 layers, of which only the convolutional and fully connected layers are computed; pooling, normalization, and activation functions are not computed. In the last three convolutional blocks, dense multi-branch convolution is used in this study. The first convolutional layer is used to partition the input features into low-dimensional features by reducing the number of channels of the feature map to 32 by 1× 1 convolution, the second convolutional layer is a conventional 3 × 3 convolution used for feature extraction, and the third convolution is used to aggregate the low-dimensional features from different branches to convert the number of channels of the features to the same dimensions as the input. The final output of the module consists of the output of the third convolutional layer and the input feature map of the first convolutional layer, spliced together to realize a dense concatenation.

**Supplementary Table 2.** Training configuration.

| **Model** | | **Parameter** | **Value** |
| --- | --- | --- | --- |
| Classification model for spikes | Backbone Network | | ResNet |
|  | Optimizer | | SGD |
|  | Initial learning rate | | 0.5 |
|  | Batch size | | 20 |
|  | Epochs | | 160 |
|  | Momentum | | 0.9 |
|  | Weight decay | | 0.0005 |
|  | Input image size | | 224 × 224 |
|  | Number of classes | | 2 (positive / negative) |
|  | Hardware | | NVIDIA GTX 2080Ti GPU |
| Classification model for fluorescence images | Backbone Network | | ResNeXt |
|  | Optimizer | | SGD |
|  | Initial learning rate | | 0.001 |
|  | Batch size | | 4 |
|  | Epochs | | 50 |
|  | Momentum | | 0.9 |
|  | Weight decay | | 0.0005 |
|  | Loss function | | Cross Entropy |
|  | Input image size | | 1024 × 1024 |
|  | Number of classes | | 4(MN/IgAN/DN/Negative) |
|  | Hardware | | NVIDIA GTX 2080Ti GPU |
| Segmentation model for electron-dense deposits | Backbone Network | | U-Net |
|  | Optimizer | | Adam |
|  | Initial learning rate | | 0.01 |
|  | Batch size | | 2 |
|  | Epochs | | 150 |
|  | Momentum | | 0.99 |
|  | Weight decay | | 0.00003 |
|  | Loss function | | Cross Entropy + Dice loss |
|  | Input image size | | 512 × 512 |
|  | Hardware | | NVIDIA GTX 2080Ti GPU |

**Supplementary Table 3.** Confusion matrix.

| Ground Truth | Prediction | |
| --- | --- | --- |
|  | Positive | Negative |
| Positive | TP | FN |
| Negative | FP | TN |

In particular, for TP (true positives): the classifier predicts a positive result that is actually positive; that is, the number of positive samples correctly identified. FP (false positive): the classifier predicts positive samples that are actually negative; that is, the number of false negative samples. TN (true negative): The classifier predicts a negative sample that is actually negative; that is, the number of negative samples that are correctly identified. FN (false negative): the classifier predicts negative samples that are actually positive; that is, the number of positive samples that are underreported.

**Supplementary Table 4.** Results of spike classification ablation experiment.

| **Module** | | **Precision (%)** | **Recall (%)** | **F1(%)** |
| --- | --- | --- | --- | --- |
| **Contextual self-attention** | **Cross-convolution** |  |  |  |
| - | + | 90.2 | 75.41 | 82.14 |
| + | - | 91.59 | 80.33 | 85.59 |
| **+** | **+** | **91.74** | **81.97** | **86.58** |

From the data in the table, it can be seen that the network with contextual self-attention improves precision, recall, and F1 by 1.54%, 6.56%, and 4.44%, respectively, compared to the model improved by adding cross-convolution only. Similarly, the addition of cross-convolution improves precision, recall, and F1 by 0.15%, 1.64%, and 0.99%, respectively, compared to the model improved by adding contextual self-attention only. However, the improvement resulting from the addition of cross-convolution is less pronounced than that resulting from the addition of contextual self-attention, indicating that contextual self-attention plays a significant role in improving the model's classification performance.

**Supplementary Table 5.** Results of electron dense ablation experiment.

| **Methods** | | **Dice (%)** | **IOU (%)** |
| --- | --- | --- | --- |
| Multiscale | Attention |  |  |
| Baseline | | 80.5 | 69.1 |
| + | - | 82.8 | 71.8 |
| - | + | 81.7 | 70.5 |
| **+** | **+** | **85.6** | **75.9** |

As shown in the table, the Dice coefficient and IOU coefficient increased by 2.3% and 2.7%, respectively, after the addition of multiscale connectivity, and the use of channel attention alone increased the Dice coefficient and IOU coefficient of the baseline model by 1.2% and 1.4%, respectively. The simultaneous application of multiscale connectivity and channel attention increased the Dice and IOU coefficients of the baseline model by 5.1% and 6.8%, separately. This implies that both multiscale connectivity and channel attention contribute to the segmentation performance of the network model, and the combination of multiscale connectivity and channel attention significantly improves the segmentation accuracy.

**Supplementary Methods 1: Model formula**

1. Multiscale module：

The input feature calculation formula of the multi-scale module is as follows:

$$X_{de}^{i}=X_{de}^{i+1}+A\left[ \sum_{j=0}^{7} {DU}^{j}\left( X_{en}^{j} \right) \right] （1）$$

$$\mathrm{DU}^{j}\left( X_{en}^{j} \right)=\left\{ \begin{aligned} ReLu\left( BN\left( Conv\left( MaxPool\left( X_{en}^{j} \right) \right) \right) \right) \\ ReLu\left( BN\left( Conv\left( Bilinear\left( X_{en}^{j} \right) \right) \right) \right) \end{aligned} \right. （2）$$

Among them, *i* and *j* represent the hierarchical levels within the range of [0,7], the function A (⋅) represents the channel attention, and DU (⋅) consists of pooling or bilinear interpolation, batch normalization, and ReLU activation functions, which are used to match the size of the feature maps between different levels.

1. Channel attention:

Channel attention is mainly achieved through two steps: squeezing and activation.

First, by contracting the feature combination, the channel descriptor 𝑧 is obtained, where the value of the 𝑐 channel is:

$$z_{c}=F_{avgp}\left( u_{c} \right)=\frac{1}{H\times W}\sum_{i=1}^{H} \sum_{j=1}^{W} u_{c}\left( i,j \right) （3）$$

Where *u* represents the feature combination of skip connections and 𝐻 × 𝑊 represents the spatial dimension.

Secondly, activation: A nonlinear gating mechanism is implemented by using two fully connected layers and the activation functions of ReLU and Sigmoid to generate the attention weight s:

$$s=W_{1}\delta\left( W_{2}z \right) （4）$$

Which 𝛿 ReLU operator, says 𝑊_1_ ∈ $R^{{C\times C}/2}$，𝑊_2_ ∈ $R^{C/{2\times C}}$ respectively are the two full connect layer has a weight.

The final output is the weighted feature map. After strengthening the important channel information, it is input into the subsequent network layer.

1. Contextual self-attention：

Contextual self-attention spatially convolution all adjacent keys within a 3 × 3 grid to contextualize each key representation. Learn the context of key $K^{1}\in R^{H\times W\times C}$ nature reflects the local neighbor key between static contextual information, we will $K^{1}$ as input 𝑋 static context. Contextual key $K^{1}$ and query 𝑄 connect, through two consecutive 1 x 1 convolution 𝐴 attention matrix ($W_{\theta}$ ReLU activation function, $W_{\delta}$ no activation functions), as follows:

$$A=\left[ K^{1} ,Q \right]W_{\theta}W_{\delta} (5)$$

To matrix according to context 𝐴 aggregate values 𝑉 to calculate characteristic figure $K^{2}$. Because $K^{2}$ to capture the dynamic characteristics of the interaction between the input, so named $K^{2}$ input of dynamic context.

$$K^{2}=V*A （6）$$

Ultimately, the output of contextual self-attention 𝑌 is a fusion of static context $K^{1}$ and dynamic context $K^{2}$.

1. Cross-convolution：

The cross-convolution consists of two decomposed asymmetric filters, $K_{1\times m}$ and $K_{m\times1}$, with receptive fields of 1 × 𝑚 and 𝑚 × 1, respectively. Let the input feature and output feature be $F_{\mathrm{in}}^{Conü}$ and $F_{\mathrm{out}}^{Conü}$, respectively, then we have:

$$F_{\mathrm{out}}^{\mathrm{Conv}}=K_{1\times m}\otimes F_{\mathrm{in}}^{\mathrm{Conv}}+K_{m\times1}\otimes F_{\mathrm{in}}^{\mathrm{Conv}}+b （7）$$

Where ⊗ represents the convolution process and 𝑏 represents the bias term.

1. Multi-branch convolution:

The expression of the network block of multi-branch convolution can be defined as follows:

$$y=x+\sum_{i=1}^{c} \mathcal{T}_{i}\left( x \right) （8）$$

Where 𝑦 is the output of the block, and 𝒯i(𝑥) can be any function that projects 𝑥 into a low-dimensional embedding for transformation, 𝐶 represents the number of transformations to aggregate, known as the cardinality, which is the extended new dimension.

1. Dense connected：

In a dense join, the network at the 𝐿 layer has 𝐿 (𝐿 + 1)/2 connections, and the dense join blocks concatenate features during feature fusion. Suppose the networks of the 𝐿 layer form a network block, 𝑥0 is the input of the network block, 𝑥l is the output of the 𝑙 layer network, and H_1_(∙) represents the mapping function of the 𝑙 layer network. Then the densely connection can be expressed as:

$$x_{1}=H_{1}\left( \left[ x_{0},x_{1},...,x_{l-1} \right] \right) (9)$$

Where [𝑥_0_, 𝑥_1_,..., 𝑥_l-1_,] represents the concatenation of features from layer 0 to 𝑙-1.

**Supplementary Methods 2: Stage-wise performance and annotation variability analysis.**

To evaluate the model’s generalization ability for deposit segmentation across different stages of MN, one-way analysis of variance (ANOVA) was performed to compare Dice and IoU scores based on pathological stages (I–IV). All statistical analyses were conducted using R software (version 4.5.0).

**Supplementary Result: Model generalization across MN stages and resistance to annotation inconsistency.**

The model demonstrated stable segmentation performance across all MN pathological stages (I–IV), with no statistically significant differences observed in Dice (p = 0.90) or IoU (p = 0.88) scores (Supplementary Figures 4B and 4C). These findings indicated consistent generalizability of the model regardless of disease stage progression.
